# New insights into the salt-responsive regulation in eelgrass at transcriptional and post-transcriptional levels

**DOI:** 10.3389/fpls.2025.1497064

**Published:** 2025-02-06

**Authors:** Huan Zhao, Xu Dong, Dazuo Yang, Qingchao Ge, Peng Lu, Chang Liu

**Affiliations:** ^1^ College of Fisheries and Life Science, Dalian Ocean University, Dalian, China; ^2^ Key Laboratory of Marine Bio-resources Restoration and Habitat Reparation in Liaoning Province, Dalian Ocean University, Dalian, China; ^3^ Department of Nursing, Zibo Vocational Institute, Zibo, China

**Keywords:** *Zostera marina*, salt stress response, miRNA, transcription factor, comparative transcriptome, degradome sequencing, gene regulation

## Abstract

**Introduction:**

The adaptation mechanisms of marine plants to the environments have garnered significant attention in recent years. Eelgrass (*Zostera marina*), a representative marine angiosperm, serves as an ideal model for investigating the mechanisms underlying salt tolerance.

**Methods:**

This study integrated mRNA, sRNA, and degradome sequencing data to identify key genes associated with salt tolerance in eelgrass.

**Results:**

The results indicate that a series of genes involved in biological processes such as “in response to water deprivation” and “biosynthesis of secondary metabolites” respond to salt stress. Analysis of cis-regulatory elements and expression similarities suggests that the ABA synthase 9-cis-epoxycarotenoid dioxygenase (NCED) may be regulated by ERF members, while phenylalanine ammonia-lyase (PAL) may be regulated by MYB members. At the post-transcriptional regulation level, miRNA156 and miRNA166 might be involved in the response by regulating potential target genes, such as members of the WRKY and HD-ZIP families. Additionally, eelgrass exhibits unique responses to salt, such as the up-regulation of genes involved in the “fucose biosynthetic process”. These findings enhance our understanding of how eelgrass adapts to the marine environment.

**Discussion:**

As a marine monocotyledon, eelgrass is helpful to find conserved salt tolerance mechanisms by cross-species comparison. By examining the transcriptional responses of homologous genes in eelgrass, rice, and maize, we identified several groups of genes that are conserved in their response to salt stress. These conserved gene resources may provide targets for genetic engineering to improve the salt tolerance of crops.

## 1 Introduction

Seagrass, a widely distributed coastal angiosperm, plays a crucial role in marine ecosystem functioning and global carbon sequestration (Ma X. et al., 2021). In order to adapt to the marine environment, seagrass has evolved different morphological (e.g. osmotic capacities), ultrastructural (e.g. absence of the real cuticle) and physiological characteristics (e.g. polyanionic, low-methylated pectins and sulfated galactans in cell walls) to tolerate salinity (Olsen et al., 2016; Wissler et al., 2011; Sandoval-Gil et al., 2023). Hence, salinity is considered as one of the major factors that condition the distribution, ecology and biology of seagrasses. Changes in salinity have the potential to impact on growth and biomass production of seagrass (Sandoval-Gil et al., 2023). In recent years, salinity levels in some seagrass beds are frequently influenced by extreme climate events such as continuous evaporation under high temperature or water exchange by rainfall (Koch et al., 2007; Tomasello et al., 2009), as well as human activities including the discharge from desalination factories (Cambridge et al., 2019), which are seriously threatened the maintenance of seagrass beds.

Eelgrass (*Zostera marina*), a member of the Angiosperms within the Monocotyledon group, is widely distributed in the coastal areas of the Northern Hemisphere (Zhang Y. et al., 2023). It is considered as one of the marine ecological model species for revealing adaptation to marine life. The activities of Na+/H+ exchangers in the plasma membrane of eelgrass have been involved in salinity tolerance (Rubio et al., 2011). Nitric oxide (NO) is also reported to play a crucial role in regulating the salinity tolerance of eelgrass by enhancing antioxidant defense mechanisms, as well as by modulating osmotic balance and energy metabolism (Wang X. et al., 2024). The analysis of the genome has revealed eelgrass exhibits some cell wall characteristics similar to those of macroalgae, which facilitate the retention of water and ions (Olsen et al., 2016). Genome-wide analysis in eelgrass revealed a reduced number of aquaporins and higher expression of plasma membrane intrinsic proteins (PIPs) compared to terrestrial plants (Shivaraj et al., 2017). These studies have revealed many genomic changes responsible for marine physiological adaptations, particularly regarding salinity tolerance.

Transcription factors (TFs) could activate or inhibit the transcription of target genes by binding specific DNA sequence (Yoon et al., 2020). Stress-responsive TFs such as the members of AP2/ERF and ARF, control the gene transcription in abscisic acid (ABA) and jasmonic acid (JA) signaling which are the key stress hormones in plant response to abiotic stress. Numerous studies have highlighted the regulatory functions of those two stress-responsive TFs in mediating terrestrial plants’ responses to salinity. For instance, *ERF106MZ* in rice or *EREB57* in maize are reported to mediate ABA signaling under salinity stress (Chen et al., 2023; Zhu et al., 2023). The salt tolerance role of *ARF5* in sweet potato has been functionally verified in transgenic *Arabidopsis thaliana* and is associated with ABA signaling (Kang et al., 2018). However, in many species, ARFs are down-regulated at high salinity, suggesting that members of this family may play a negative role (Ye et al., 2020; Wang C. et al., 2024). Besides, other TFs including MYB, NAC, bHLH and WRKY, are also predicted to be differentially expressed under salinity stress. For example, the *AtMYB20* in *A. thaliana* is up-regulated by salt stress, and transgenic plants overexpressing *AtMYB20* showed improved tolerance to salt stress (Cui et al., 2013). The heterologous overexpression of strawberry *FaMYB63* in *A. thaliana* increased salt tolerance by reactive oxygen species (ROS) clearance (Wang S. et al., 2024). *SlNAC4* in *Suaeda liaotungensis* was also verified to enhance tolerance to salt by regulating ABA metabolism through heterologous overexpressed (Liu et al., 2023). *ThNAC13* in *Tamarix hispida* was proved to improve salt tolerance by binding to *ThPP2* gene to enhance antioxidant enzyme activity (Liu R. et al., 2024). *MYC2*, a bHLH family member in *A. thaliana* and *BpWRKY32* in *Betula platyphylla* were verified to increase salt tolerance (Verma et al., 2020; Liu Z. et al., 2024). In summary, these TFs primarily influence salt stress response and tolerance by modulating the expression of downstream genes, which participate in enhancing the antioxidant system, maintaining ion balance, regulating proline synthesis and ABA response. However, there is a notable scarcity of studies investigating these regulatory factors in seagrass species.

Plant microRNAs (miRNAs), typically consisting of 20-24 nucleotides, are also important post-transcriptional regulatory molecules (Begum, 2022). miRNAs could influence the expression of target genes. The function of stress-responsive miRNAs and their target under salt stress have been studied in model plants and some economic terrestrial crops. The expression of Tch-miR167 was negatively correlated to the expression of *TcARF6*, which indicated that *TcARF6* increases salt tolerance in *Tamarix chinensis* by post-transcriptional regulation (Ye et al., 2020). The miR393 in *A. thaliana* regulated the salt-responsive pathway by scaffold protein RACK1A mediated ABA signaling pathways (Denver and Ullah, 2019). Overexpression of Osa-miR319a in transgenic creeping bentgrass *Agrostis stolonifera* inhibited the expression of *AsPCF6*, *AsPCF8*, *AsTCP14*, and *AsNAC60*, and modified the salt tolerance of transgenic plant (Zhou et al., 2013). The ABA receptor genes *NtPYL2* and *NtSAPK3* showed up-regulated expression in TaemiR408 tobacco lines, which indicated that TaemiR408 is associated with the miRNA modulation for ABA signaling to improve salt tolerance (Bai et al., 2018). miR172a and miR172b in cereal crops, miR398 in tomato and miR528-AO in rice were also proved to play a regulatory role in maintaining ROS homeostasis during salt stress (Cheng et al., 2021; He et al., 2021; Wang et al., 2021). In contrast, research on the role of miRNAs in aquatic plants under environmental stress conditions remains limited. Studies addressing the response of microRNAs to salt stress have been reported in species such as *Dunaliella salina* (Gao et al., 2019) and *Spirulina platensis* (Zhao et al., 2016). Some miRNAs in eelgrass have been identified by genomic analysis by Olsen et al. (2016) and Ma et al. (2021), which provide information for further research on the regulation mechanism of miRNA under abiotic stress.

The function of TFs and microRNAs in terrestrial plants have been identified as important regulatory elements that enable plants to withstand salt stress. Compared to terrestrial plants, the transcriptional and post-transcriptional response of those regulator in seagrass under stress remain elusive. Previous studies on eelgrass exhibited that high salinity not only inhibits seed germination but also effect the growth rate and survival of eelgrass (Zhang Y.-H. et al., 2023; Zhang Y. et al., 2023). In order to investigate the molecular mechanism and identify the potential regulatory factors, we performed a comprehensive analysis on the transcriptional response of eelgrass to salt stress using mRNA sequencing and small RNA sequencing data. Based on differential expression characteristics and similarity in expression trends, we identified various TFs, miRNAs, and functional target genes that may be associated with salt tolerance in eelgrass. The relationship between target genes and miRNAs was predicted and proved by degradome sequencing. This discovery provides more information about the molecular mechanisms underlying the adaptation of eelgrass to hypersalinity. Furthermore, we chose two terrestrial economic crops, rice and maize which also belong to Monocotyledon, to compare the homologous differential expression genes (DEGs) in response to salt stress. The comparative analysis not only sheds light on the conserved and specific mechanism in eelgrass adaption to hypersalinity but also provides gene resources for functional verification in terrestrial model species.

## 2 Materials and methods

### 2.1 Plant and sampling

The eelgrass used in this study was sourced from a seedling factory in Qingdao, Shandong Province of China. Initially, the plants underwent a cleaning process and were then methodically arranged within a temporary tank. The base of the temporary storage tank comprised sand and stone that had undergone high-temperature sterilization, with the filtered seawater. The seawater was maintained with temperature of 17.5°C and salinity of 30, while the light intensity was approximately 120 µmol/(m²·s). Following three days of cultivation, the plants exhibiting robust growth were selected and transferred to experimental boxes No. 1 and No. 2, with seawater salinities of 30 and 50 (Xu et al., 2015), respectively. After 12 hours of treatment, the plants were removed from the boxes and promptly placed in liquid nitrogen for rapid freezing. A total of 6 plants were sampled in each treatment to build three biological replicates (with 2 plants being mixed to form a sample) for small RNA and mRNA sequencing. Different tissues were separated with sterile scissor. As the individual root samples did not provide enough material for sequencing, we mixed the stem and root together as one tissue (SR tissue). Besides, another 3 plants under high salt conditions were collected as a mixed sample for degradome sequencing.

### 2.2 RNA-seq library construction and sequencing

The total RNA was extracted using the RNAprep Pure Plant Kit DP441 (Tiangen, Beijing, China) according to the kit instructions. Then, mRNA was purified from total RNA using poly-T oligo-attached magnetic beads. Fragmentation was carried out using divalent cations under elevated temperature in First Strand Synthesis Reaction Buffer. First strand cDNA was synthesized using random hexamer primer and M-MuLV Reverse Transcriptase (RNase H-). Second strand cDNA synthesis was subsequently performed using DNA Polymerase I and RNase H. Remaining overhangs were converted into blunt ends via exonuclease/polymerase activities. After adenylation of 3’ ends of DNA fragments, Adaptor with hairpin loop structure were ligated to prepare for hybridization. In order to select cDNA fragments of preferentially 370-420 bp in length, the library fragments were purified with AMPure XP system. Then PCR was performed with Phusion High-Fidelity DNA polymerase, Universal PCR primers and Index (X) Primer. At last, PCR products were purified. After the library construction is completed, preliminary quantification is carried out using Qubit, and the library is diluted to 1.5ng/ul. Subsequently, the inserted fragments of the library are detected. Once the inserted fragments meet expectations, the effective concentration of the library is accurately quantified using realtime PCR to ensure the quality of the library. The qualified libraries were pooled and sequenced on Illumina sequencing platforms, according to effective library concentration and data amount required.

### 2.3 Small RNA library construction and sequencing

The total RNA extracted was also used for small RNA sequencing (Zhang M. et al., 2023; Hu et al., 2021). We used NEB Next^®^ Multiplex Small RNA Library Prep Set (NEB E7300L) for small RNA library construction. The 5 ‘end of small RNA has a complete phosphate group and the 3’ end of small RNA has a hydroxyl group. So special 3’ and 5’ adaptors were ligated to the 3’ and 5’ ends of small RNA based on the special structure of the 3’ and 5’ end of small RNA, respectively. Following this step, the first strand cDNA was synthesized subsequent to hybridizing with the reverse transcription primer. Subsequently, the double-stranded cDNA library was created via PCR enrichment. The libraries containing insertions ranging from 18 to 40 bp were then readied for sequencing after purification and size selection. Upon completion of the library construction process, the inserted fragments were quantified and the effective concentration (9~14.41 nmol/L) was assessed to ensure the quality of the libraries. The qualified libraries were pooled and sequenced on Illumina sequencing platforms, according to effective library concentration and data amount required. To gather more data for analysis, additional sequencing runs were conducted on four small RNA samples (L30-2, L30-3, L50-2, L50-3), which were collectively labeled as “batch2”.

### 2.4 RNA-seq data analysis

The RNA-seq data was subjected to quality assessment using Fastqc and then preprocessed using trim-galore v0.6.10. Basic data information, such as sequence number and sequence length, was obtained using the seqkit v2.1.0. The preprocessed data was then aligned to the reference genome Zostera marina v3.1 from Phytozome13 using hisat2 v2.2.1. The results were sorted using samtools, and the quantification of gene loci was performed with featureCounts v2.0.6. Additionally, stringtie v2.2.2 was utilized to compute Fragments Per Kilobase of transcript per Million mapped reads (FPKM). Additionally, the sample R50-1 was excluded from further analysis due to its low mapping rate. Besides, we analyzed the PRJNA342750 data from NCBI related to the NaCl response.

The DESeq2 R package was used to analyze gene expression differences based on read count data, with the criteria for DEGs screening set as false discovery rate (FDR) ≤ 0.05 and fold change (FC) ≥ 2. The identified DEGs were classified according to their differential expression in different tissues, and the R package ComplexHeatmap was utilized to draw heat maps. The gene annotation was downloaded from Phytozome13, and complemented by eggNOG and KASS for GO and KEGG annotation. Mercator4 was utilized for mapman annotation of genes. Functional enrichment analysis was performed using the R package clusterProfiler (FDR ≤ 0.05).

### 2.5 Construction of gene co-expression network in eelgrass

To reveal the transcriptional regulatory network of eelgrass in response to high salt stress, we first conducted co-expression network analysis using the Weighted Gene Co-expression Network Analysis (WGCNA) package. Our dataset was combined with 58 additional samples obtained from the NCBI database (refer to **Supplementary Data S2**). In the initial stages of data preprocessing, we utilized Sratoolkit for data format conversion and Trim-galore for the identification and removal of adaptors and low-quality reads. Subsequently, the preprocessed data were aligned to a reference genome using hisat2. RSeQC was employed to ascertain the strand specificity of the mapped data. Following this, Stringtie was employed to compute the gene expression levels in fragments per kilobase of transcript per million mapped reads (FPKM), with genes exhibiting a Coefficient of Variation (CV) ≥ 1 retained for further analysis. Then, the gene expression values were log2-transformed (log2[FPKM+1]) for normalization. To construct an appropriate correlation weighting value (soft threshold) for co-expression network prediction, we utilized the pickSoftThreshold function in the R package WGCNA, with a criterion of SFT.R.sq ≥ 0.85 to ensure a scale-free network structure. Subsequently, we determined the soft threshold value of 8 for network construction. The one-step method (blockwiseModule function, parameters: networkType=“signed hybrid”; minModuleSize=30; mergeCutHeight=0.25) was then employed to build the network, and eigengene analysis was applied to illustrate the gene expression trends within each module. Finally, we used the R package Complex Heatmap to generate heat maps to visualize the distribution of DEGs in co-expression modules.

### 2.6 Identification of microRNAs and DEmiRNAs

Small RNA data were aligned to the reference genome Zostera marina v3.1. The mapping files were merged to identify microRNAs using ShortStack v3.8.5 and miRDeep-P2 v1.1.4. Subsequently, bedtools v2.30.0 was employed to distinguish the same and unique MIRNA loci predicted by the two tools. The non-coding RNAs such as rRNA, tRNA, snRNA, and snoRNA were excluded from this analysis. For the identification of these non-coding RNAs, distinct prediction tools including barrnap, tRNAscan-SE, and Infernal-Rfam were utilized.

The pre-miRNA sequences were extracted based on the predicted MIRNA loci. Then, RNAfold (https://www.tbi.univie.ac.at/RNA/tutorial/) was used to examine the secondary structure of pre-miRNAs. Simultaneously, the predicted mature miRNA sequences were compared with those documented in miRbase (http://www.mirbase.org/) to determine miRNA families based on similarity. Meanwhile, we compared the MIRNA and gene location to classify the MIRNA into two types: the intragenic ones and the intergenic ones.

The read counts for miRNA and other sRNA loci were calculated using the ShortStack. According to the correlation analysis on the expression of small RNAs, we removed four samples in subsequent analysis, including L30.1, L50.1, SR30.3, and SR50.2. The R package EdgeR was used for counts per million (CPM) quantification. Differentially expressed miRNAs (DEmiRNAs) were identified using both EdgeR and DESeq2, with a threshold of p-value ≤ 0.05. The online tool psRNATarget (2017 release) was employed to predict miRNA target genes.

### 2.7 qRT-PCR detection of DEGs and DEmiRNAs

We utilized the SteadyPure Plant RNA Extraction Kit (Accurate Biotechnology, Changsha, Hunan, China) for mRNA extraction and the Evo M-MLV Reverse Transcription Kit (Accurate Biotechnology, Changsha, Hunan, China) for reverse transcription of RNA. For small RNA extraction, we utilized the SteadyPure Tissue and Cell Small RNA Extraction Kit (Accurate Biotechnology, Changsha, Hunan, China) followed by reverse transcription using the miRcute Enhanced miRNA cDNA First Strand Synthesis Kit (Tiangen, Beijing, China). The procedures were conducted according to the instructions. Quantitative real-time PCR (qRT-PCR) experiments were carried out using 7500 Fast Real-Time PCR equipment, with the following thermal cycling conditions: 95°C for 30s, followed by 40 cycles at 95°C for 5s and 60°C for 30s. In total, 9 DEGs and 4 DEmiRNAs were verified. The primers for these genes and miRNAs are listed in **Supplementary Data S12**. For normalization, *18S rRNA* and *U6* served as the reference gene, and relative expression changes were calculated using the 2^−ΔΔCt^ method. The statistical analysis was performed using the function t.test in R software.

### 2.8 Degradome sequencing and analysis

Total RNA was isolated and purified using TRIzol reagent (Invitrogen, Carlsbad, CA, USA) following the manufacturer’s procedure. The RNA amount and purity of each sample were quantified using NanoDrop ND-1000 (NanoDrop, Wilmington, DE, USA). The RNA integrity was assessed by Agilent 2100 with RIN number >7.0.

Poly(A) RNA is purified from plant total RNA (20μg) using poly-T oligo-attached magnetic beads using two rounds of purification. Because of the 3′ cleavage product of the mRNA contains a 5′-monophosphate, the 5’ adapters were ligated to the 5’ end of the 3′ cleavage product of the mRNA by the RNA ligase. The next step is reverse transcription to make the first strand of cDNA with a 3′-adapter random primer and size selection was performed with AMPureXP beads. Then the cDNA was amplified with PCR by the following conditions: initial denaturation at 95°C for 3 min; 15cycles of denaturation at 98°C for 15 s, annealing at 60°C for 15 s, and extension at 72°C for 30 s; and then final extension at 72°C for 5 min. The average insert size for the final cDNA library was 200-400 bp. At last, we performed the 50bp single-end sequencing on an Illumina Hiseq 2500 (LC Bio, China) following the vendor’s recommended protocol.

Degradome sequencing data were analyzed using ACGT101-DEG (LC Sciences, Houston, Texas, USA), which incorporates CleaveLand4. The analysis began with the preprocessing of sequences and followed by the creation of degradome density file. The miRNA sequences were aligned with the reference transcript sequence to identify potential clipping sites. Finally, the integration of sequencing data and comparison results allowed for the identification of targets and cutting sites which are supported by evidence of degradation fragments, and obtain the P-values.

### 2.9 Prediction of cis-regulatory elements and corresponding TFs

The 1000 bp upstream sequence of DEGs and the 2000 bp upstream sequence of MIRNA loci were extracted, followed with cis-regulatory element prediction carried out using FIMO v4.11.2 (parameter: default) with the JASPAR2024_CORE_non-redundant database. The classification of cis-regulatory elements was based on the prediction results obtained from plantTFDB for the corresponding proteins documented in the JASPAR database. At the same time, we used the plantCARE online tool to make additional predictions.

The TFs of eelgrass were predicted using the Plant Transcription Factor Database (plantTFDB) available at http://planttfdb.cbi.pku.edu.cn/. The expression patterns of these TFs were compared with those predicted target genes. TFs with a Pearson correlation coefficient (PCC) of 0.8 or higher were selected as candidate regulators. Cytoscape v3.10.2 (https://cytoscape.org/) was employed for visualization of the transcriptional regulatory network.

### 2.10 Gene family analysis

Based on the results of functional enrichment analysis in eelgrass and references to genes involved in salt tolerance in terrestrial plants, we selected a set of genes for gene family analysis.

Aquaporins in eelgrass containing PF00230 domains were identified by hmmscan 3.4 (parameter:-E 10^-5^). Four classes of ion channel proteins, the NHX, KEA, and CHX family genes were gathered from the research of Olsen et al. (2016). Blast comparison was performed based on the protein sequences to find the corresponding gene IDs in Zostera marina v3.1. Additionally, genes possessing the PF01699 domain were classified as members of the Ca^2+^/H^+^exchanger antiporter (CAX) family using the hmmscan tool with a parameter setting of -E 10^-5^. Antioxidant enzyme genes, including superoxide dismutase (SOD), catalase (CAT), and glutathione peroxidase (GPX) collected based on the research of Olsen et al. (2016). Blast comparisons were carried out using the protein sequences to determine the corresponding gene IDs in Zostera marina v3.1. Moreover, a list of ascorbate peroxidase (APX) family genes from *A. thaliana* was retrieved from NCBI, and the homologous genes in eelgrass were identified using OrthoFinder. Moreover, OrthoFinder was utilized to identify homologous genes in eelgrass based on hormone-related genes in *A. thaliana* summarized by Altmann et al. (2020). Genes involved in the salt overly sensitive (SOS) pathway were identified by first compiling a list of *A. thaliana* genes associated with this pathway (Sandoval-Gil et al., 2023). Subsequently, using the *A. thaliana* gene IDs, the ortherFinder identification results were utilized to locate the corresponding genes in eelgrass.

Using protein sequences, we employed blastp v2.12.0 (parameters: -evalue 10^-10^, -max_target_seqs 5) to identify the top five homologous genes in *A. thaliana*, rice, and maize to construct phylogenetic tree. Besides, to compare the response to salinity across species, 100 rice sra data and 57 maize sra data (**Supplementary Data S9**) were downloaded from the NCBI. The reference genome was obtained from Ensembl Plants. Data preprocessing, sequence alignment, gene expression quantification, and identification of DEGs were performed following the same procedures as those for the samples of eelgrass. The GO annotation of genes in rice and maize was performed by eggNOG. To identify the set of genes that are widely up-regulated in rice and maize, we counted sample comparisons for up-regulated DEGs. Then, we selected the genes based on the top 10% percentile of comparison counts and performed functional enrichment analysis.

## 3 Results

### 3.1 The transcriptional response of genes under salt stress

The raw data of RNA-seq includes 41.8-53.4 million reads, and the mapping rate of most samples is greater than 80%. We identified a total of 409 DEGs between the samples under salinity 50 and 30 treatment using DESeq2 (**Supplementary Data S1**; **Supplementary Figure S1A**), including 188 genes from leaf tissue and 236 genes from SR tissue (the mixed tissue of stem and root). Additionally, an analysis of the PRJNA342750 dataset revealed 334 DEGs in leaf tissues in response to NaCl. There was minimal overlap among the DEGs identified across the three groups (**Supplementary Figure S1B**), indicating distinct responses to salt stress in different tissues and under varying experimental conditions.

**Figure 1:**
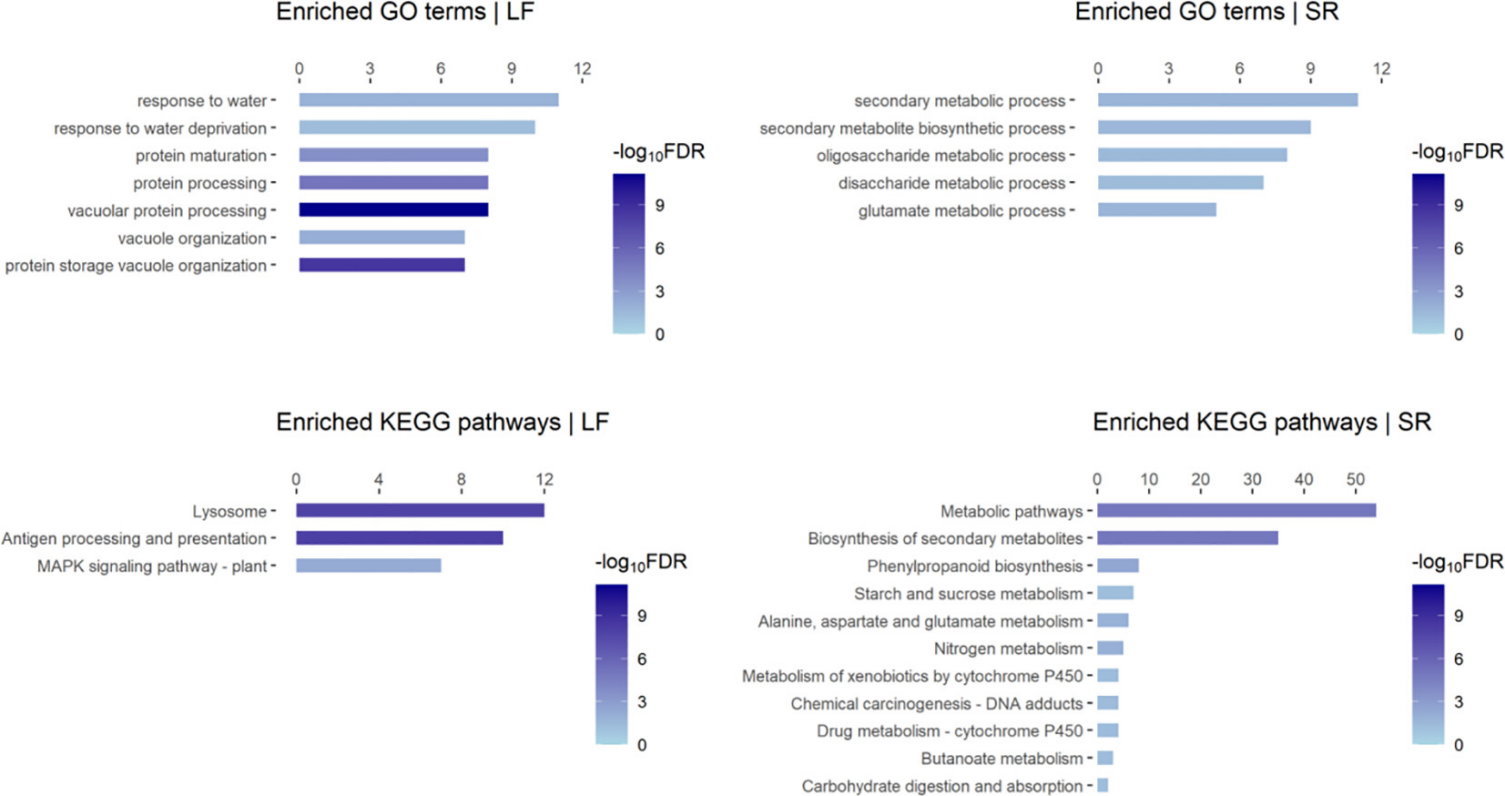
Enriched GO terms and KEGG pathways of responsive DEGs to high salt in eelgrass. “LF” refers to the leaf; “SR” refers to the mixed tissue of stem and root. The length of the bar refers to the count of DEGs in a Go-term or KEGG pathway.

We performed functional enrichment analysis on the DEGs identified in our data, which revealed several enriched GO terms and KEGG pathways such as “response to water deprivation (GO:0009414)”, “MAPK signaling pathway-plant (map04016)”, “Biosynthesis of secondary metabolites (map01110)”, “Phenylpropanoid biosynthesis (map00940)” (**Figure 1**; **Supplementary Figures S1C, D**). DEGs in GO:0009414 were predominantly up-regulated in leaf, including members of the PP2C family (*Zosma01g16060*), ERF family members (*Zosma05g33490* and *Zosma03g37150*), Late embryogenesis abundant protein (*Zosma05g03710*), and the UPF0057 family gene (*Zosma03g14850*). Conversely, DEGs in map04016 were primarily down-regulated in leaf, including family members of MAPKKK (*Zosma03g00830*, *Zosma03g00810*), ERF (*Zosma06g29270*), WRKY (*Zosma06g26970*), and CALM (*Zosma06g17200*). What’s more, DEGs in map01110 were mainly up-regulated in SR tissue, including Cinnamate 4-coumarate:CoA ligase (*Zosma01g00600*), trans-cinnamate 4-monooxygenase (*Zosma02g22230*), cinnamoyl-CoA reductase (*Zosma03g12930*), phenylalanine ammonia-lyase (*Zosma03g22300*), cinnamyl-alcohol dehydrogenase (*Zosma06g25800*), chalcone synthase (*Zosma01g25890*), and chalcone isomerase (*Zosma06g21110*). MapMan annotation and enrichment results indicated that salt stress may induce programmed cell death (**Supplementary Figure S1C**). *Zosma03g26890* is a proline dehydrogenase and down-regulated under salt stress, indicating the proline accumulation might be enhanced in eelgrass. (**Supplementary Figure S1D**). Additionally, plant hormones such as ABA and gibberellin may play a role in the response to salt stress (**Supplementary Figure S1D**).

**Figure 2:**
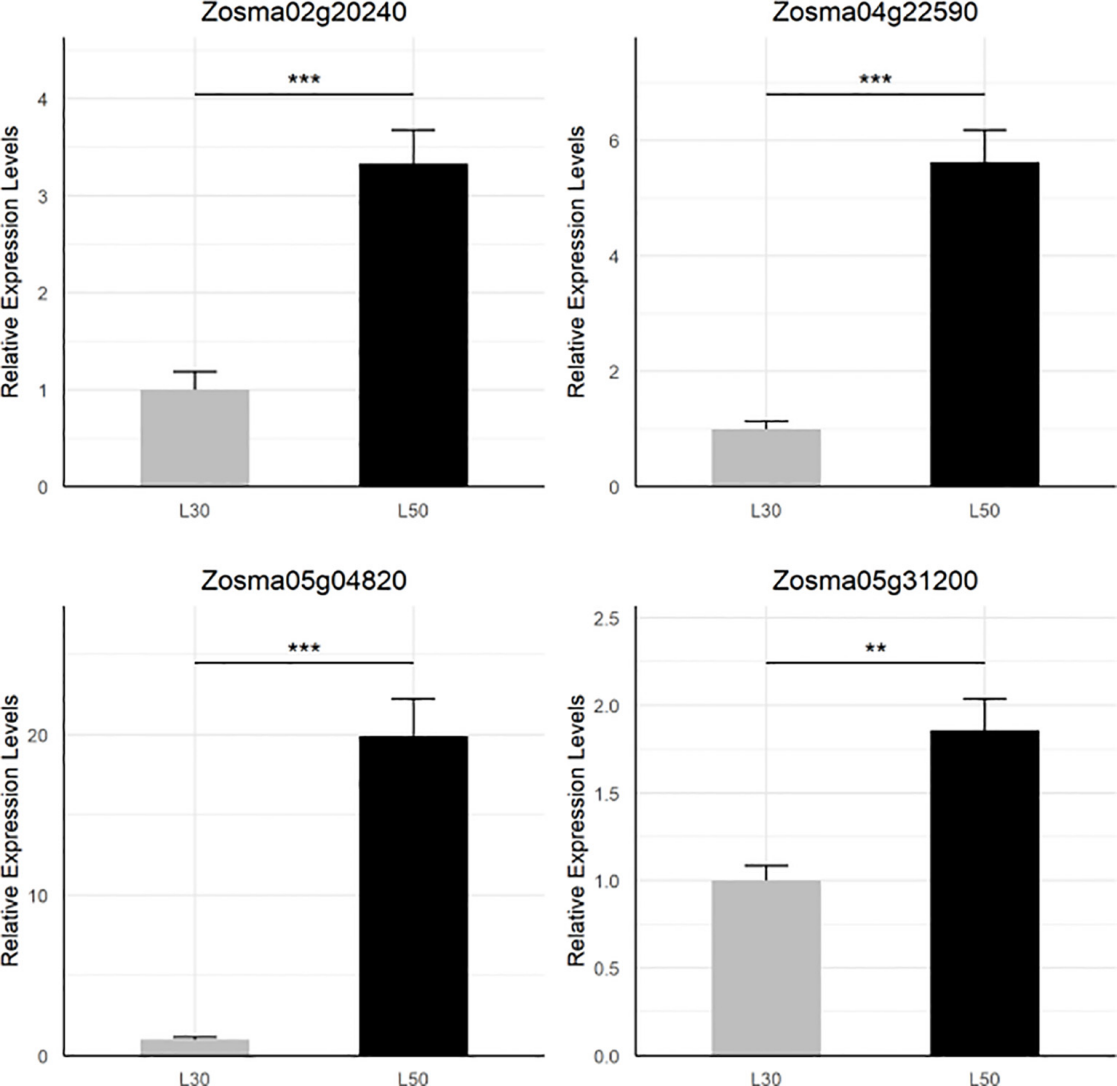
qRT-PCR validation of four DEGs that are up-regulated in all comparisons. The star marks on statistical charts represent the statistical significance: “*” stands for P < 0.05, “**” stands for P < 0.01, “***” stands for P < 0.001.

By examining the overlapping DEGs, we identified four genes (*Zosma02g20240*, *Zosma04g22590*, *Zosma05g04820*, *Zosma05g31200*) that were up-regulated in all three comparisons (**Supplementary Figure S1B**). *Zosma05g04820* is a member of the AP2/ERF family, and its homologous genes participate in abiotic stress such dehydration (Sakuma et al., 2002). *Zosma04g22590* encodes NCED is a crucial enzyme in the biosynthesis of ABA (Qin and Zeevaart, 1999), of which homologous genes function in drought tolerance (Iuchi et al., 2001). *Zosma02g20240* belongs to the CAX family of cation antiporters, of which the homologous gene is involved in calcium transport (Emery et al., 2012). *Zosma05g31200* is a senescence-associated protein, of which homologous genes are involved in various abiotic stress including salt stress (Barajas-Lopez et al., 2021). The transcriptional response of these four DEGs was further verified through qRT-PCR assays (**Figure 2**).

We observed that the tissue-specific responsive genes account for a large proportion of the DEGs identified by our data (**Supplementary Figure S1B**). Consistently, the results of functional enrichment analysis on these gene sets (**Supplementary Figure S1E**) were similar to those described above (**Figure 1**), which indicate enhanced and impaired biological processes in different tissues. Especially, we found that an enriched GO term “fucose biosynthetic process” was identified from up-regulated genes in leaf. In a previous study, fucose was found as the main component of extracted polysaccharides in *Lessonia* (Zou et al., 2019). This raises the question of whether fucose is involved in the adaptation of marine plants to high-salt environments. What’s more, we examined the specific DEGs in response to NaCl (identified by PRJNA342750 data): 118 genes were up-regulated, which were involved in biological processes such as “response to stimulus (GO: 0050896)”, “cobalamin biosynthetic process (GO: 0009236)” and “heme biosynthetic process (GO: 0006783)”; 167 genes were down-regulated, which were involved in the process such as “carbohydrate metabolic process (GO: 0005975)” and “mucilage metabolic process (GO: 0010191)”. These results help us to gain a deeper understanding of the response of eelgrass to salinity changes at the organ level.

### 3.2 Salt-responsive DEGs in co-expression modules

Utilizing our data and transcriptome data from 58 samples of eelgrass obtained from NCBI (**Supplementary Data S2**), we constructed a gene co-expression network that revealed a total of 36 gene modules, each containing at least 30 genes (**Supplementary Data S3**; **Supplementary Figures S2A, B**). The analysis (**Supplementary Figure S2C**) indicated that the up-regulated genes under salt stress in leaf were predominantly distributed in the M3, M4, M6, and M7 modules, while the down-regulated genes in leaf were primarily found in the M1, M2, M4, and M6 modules. In SR tissue, most of the up-regulated genes were distributed in the M4 and M6 modules, while the down-regulated genes were mainly distributed in the M2, M4, M6, and M7 modules.

**Figure 3:**
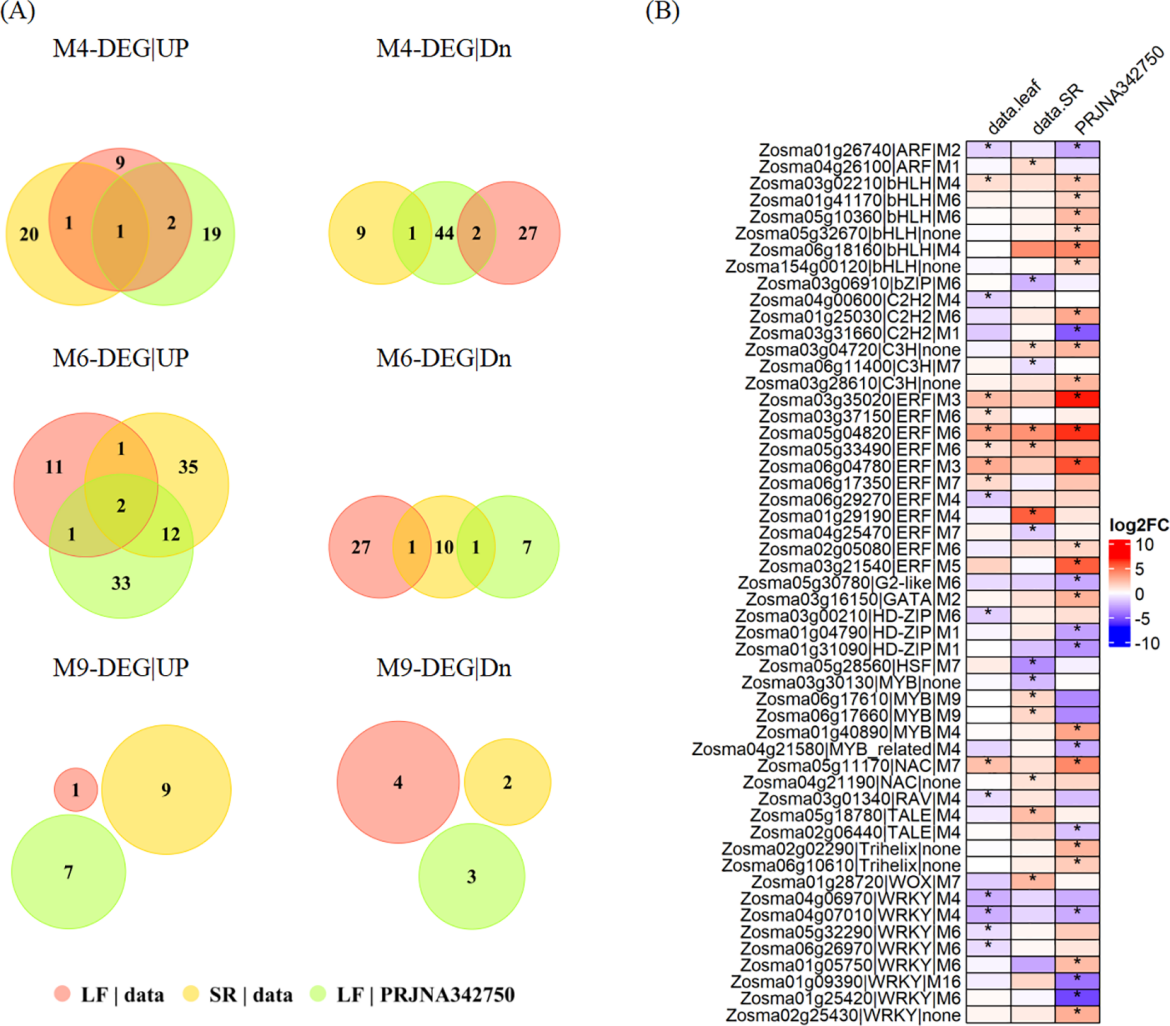
Salt-responsive DEGs in WGCNA modules. **(A)** Up-regulated or down-regulated genes in response to high salinity assigned to M4, M6 and M9. **(B)** The transcriptional response of TFs to high salinity. The asterisk stands for a significant difference in the expression comparison.

We focused on the salt-responsive DEGs in modules M4, M6, and M9 due to their expression patterns (**Figure 3A**; **Supplementary Figure S2B**): M4 exhibited a high expression primarily in leaf. M6 was notably expressed in leaf, stems, and roots. M9 showed predominant expression in stem and root. M4 harbored a number of metal transporters that were up-regulated (**Supplementary Figure S2D**), such as the mitochondrial iron transporter (*Zosma03g13600*) and several heavy metal-associated domain-containing proteins (*Zosma04g04780*, *Zosma02g05210*, *Zosma02g16380*). Conversely, many DEGs related to photosynthesis in M4 were significantly down-regulated (**Supplementary Figure S2D**), including the light-harvesting complex I chlorophyll a/b binding proteins (*Zosma02g19650*, *Zosma03g33730*, *Zosma03g35370*, *Zosma05g20560*) and the photosystem II reaction center *PSB28* protein (*Zosma03g24460*). Furthermore, several nutrition-related DEGs in leaf were up-regulated (**Supplementary Figure S2D**), including nitrite reductase (*Zosma02g18160*, *Zosma03g25160*) in M4, nitrate transmembrane transporters (*Zosma05g08330*), proton/phosphate symporters (*Zosma05g32160*) and ferritin (*Zosma02g21810*) in M6. These findings suggest a shift in the nutritional status of eelgrass under salt stress. Additionally, M6 contains a few up-regulated DEGs associated with calcium-dependent signaling (**Supplementary Figure S2D**), such as calmodulin-related calcium sensor proteins (*Zosma01g15140*, *Zosma01g36630*, *Zosma04g24750*), indicating heightened calcium signaling activity in response to high salinity. A set of up-regulated DEGs in M9 were involved in secondary metabolism (**Supplementary Figure S2D**), such as phenylalanine ammonia-lyase (*Zosma03g22300*), cinnamoyl-CoA reductase (*Zosma03g12930*) and glutamate decarboxylase (*Zosma02g19830*), indicating that the plants might undergo secondary metabolic changes in stem and root.

TFs are potential regulators for genes in co-expression modules. The M4 module contains 12 salt-responsive TFs. Three of these TFs were up-regulated in leaf, including two bHLH family members (*Zosma03g02210*, *Zosma06g18160*) and one MYB family member (*Zosma01g40890*). Two of these TFs were up-regulated in SR tissue, including one ERF family member (*Zosma01g29190*) and one TALE family member (*Zosma05g18780*). The M6 module includes 14 salt-responsive TFs. Eight of these TFs were up-regulated in leaf, including four ERF family members (*Zosma03g37150*, *Zosma05g04820*, *Zosma05g33490*, *Zosma02g05080*), two bHLH family members (*Zosma01g41170*, *Zosma05g10360*), one *C2H2* family member (*Zosma01g25030*), and one WRKY family member (*Zosma01g05750*). Two ERF family members (*Zosma05g04820* and *Zosma05g33490*) were up-regulated in SR tissue. M9 module contains 2 salt-responsive TFs, which belong to MYB family members (*Zosma06g17610* and *Zosma06g17660*) and show up-regulated expression in SR tissue. How these TFs regulate the response of eelgrass to salt stress is an issue that remains to be resolved. (**Figure 3B**; **Supplementary Figure S2E**; **Supplementary Data S4**). We analyzed the cis-regulatory elements located within the 1000 bp region upstream of DEGs. Our findings revealed that the motifs potentially binding TFs such as bHLH, ERF and MYB family members (**Supplementary Figure S2F**) are universally distributed in the promoter of DEGs. Moreover, predictions from the PlantCARE indicate that plant hormone signals, such as ABA and JA, may also contribute to the regulatory mechanisms in eelgrass (**Supplementary Figure S2G**).

### 3.3 Identification and classification of microRNAs

**Figure 4:**
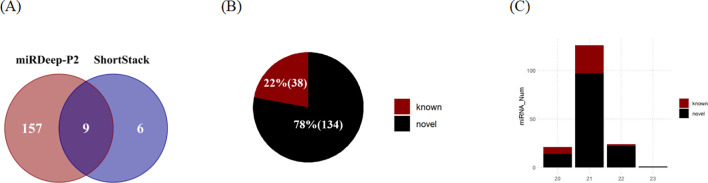
Identification of microRNAs in eelgrass. **(A)** The number of MIRNAs identified by ShortStack and miRDeep-P2. **(B)** The number of identified MIRNAs belonging to known and novel families. **(C)** The number of miRNAs (mature sequence) of different lengths.

In this study, a total of 172 MIRNA loci were identified through the analysis of small RNA sequencing data (**Figure 4A**). miRDeep-P2 exhibited a higher sensitivity compared to Shortstack, being able to identify 157 miRNAs independently. Additionally, ShortStack detected 6 miRNAs that were not identified by miRDeep-P2. Of these sites, 9 were found to be identified by both the Shortstack and miRDeep-P2 tools, which are considered the most reliable sites for MIRNA. The majority of these miRNAs were found to be located on chromosomes (**Supplementary Figure S3A**), with a few being situated on the scaffold. The length distribution of the identified mature sequences was predominantly 20-22 nt, aligning with the typical length characteristics of miRNAs in plants (**Figure 4C**).

Subsequently, a comparison was conducted between the mature sequences obtained from the predicted sites and those documented in miRbase (**Figure 4B**). This comparison led to the annotation of a total of 38 miRNAs belonging to known MIRNA families, while an additional 134 miRNAs were identified as *de novo* loci (**Supplementary Data S5**). Among the known families, *MIR156* was identified to have the highest number of loci in eelgrass, specifically containing six members. Moreover, several other families, including *MIR166*, *MIR172*, *MIR164*, *MIR396*, *MIR319*, and *MIR390*, were found to have more than two members each.

Simultaneously, the relative positions of MIRNAs to neighboring genes were assessed. A total of 41 MIRNAs were categorized as intragenic, while 131 were categorized as intergenic (**Supplementary Figure S3B**). Among the intragenic MIRNAs, 31 were located within intron regions, 3 were found in exon regions, and 7 spanned both introns and exons (**Supplementary Figure S3C**). For 78.3% of the intergenic MIRNAs were located within 20,000 bp of an upstream gene (**Supplementary Figure S3D**).

### 3.4 Salt-responsive miRNAs in eelgrass

**Figure 5:**
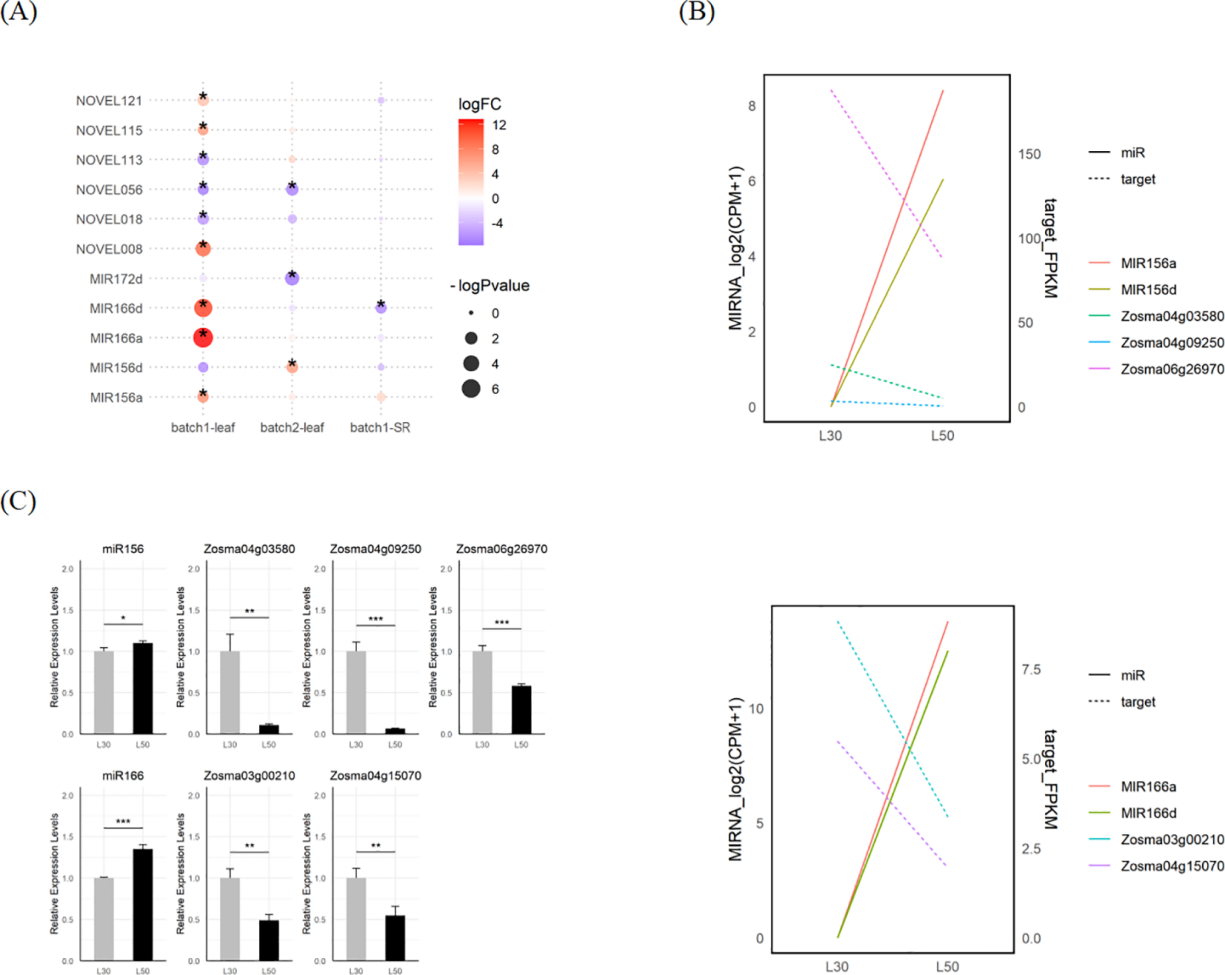
DEmicroRNAs and their potential targets in eelgrass. **(A)** The DEmiRNAs in response to salt stress in eelgrass identified by edgeR. **(B)** The expression of MIR156, MIR166 and their predicted targets in L30 and L50. **(C)** Validated expression of MIR156a/d and MIR166a/d mature sequences as well as their predicted targets using qRT-PCR. The gray bar stands for L30, while the black bar stands for L50. The star marks on statistical charts represent the statistical significance: “*” stands for P < 0.05, “**” stands for P < 0.01, “***” stands for P < 0.001.

We identified a total of 14 DEmiRNAs in response to salt stress using edgeR and DESeq2. There were 8 overlapped DEmiRNAs identified by both tools, including *MIR166a*, *MIR166d*, *MIR172d* and a series of *de novo* loci (**Figure 5A**; **Supplementary Figure S3E**; **Supplementary Data S6**). The transcriptional responses of miRNAs from these loci such as miR156, miR166 and NOVEL008 were further validated through qRT-PCR assays (**Figure 5C**; **Supplementary Figure S3F**). To uncover the potential mechanisms regulating miRNA expression, we analyzed the cis-regulatory elements within the 2000 bp upstream region of MIRNAs. The results showed that there were many potential TF binding sites upstream of the MIRNA. The binding sites for TFs such as bZIP, C2H2, and Dof are widely present (**Supplementary Figures S4A, B**). Additionally, predictions from plantCARE indicated that the upstream regions of DEmiRNAs were enriched in cis-regulatory elements associated with abiotic stress and hormone responses. It suggests that diverse signaling pathways, such as those related to osmotic stress (STRE) and JA (CGTCA-motif and TGACG-motif), play a role in regulating microRNA expression in eelgrass (**Supplementary Figure S4C**).

### 3.5 miRNA-target module in eelgrass

We employed psRNATarget to predict microRNA target genes, of which 35 were DEGs in response to salt stress and potentially targeted by DEmiRNA (**Supplementary Data S7**). There are a number of target genes whose expression changes are negatively correlated with their corresponding miRNAs, such as miR156 and miR166. Among them, *Zosma06g26970*, is a member of WRKY family and identified as a target gene for miR156; *Zosma03g00210*, is a member of the HD-ZIP family and identified as a target gene for miR166 (**Figure 5B**). Both of them exhibit down-regulated expression at high salinity, of which homologous genes has been reported to be involved in salt stress (Jiang and Deyholos, 2009; Yadav et al., 2021). The responses of these miRNAs and their target genes were further validated through qRT-PCR assays (**Figure 5C**).

**Figure 6:**
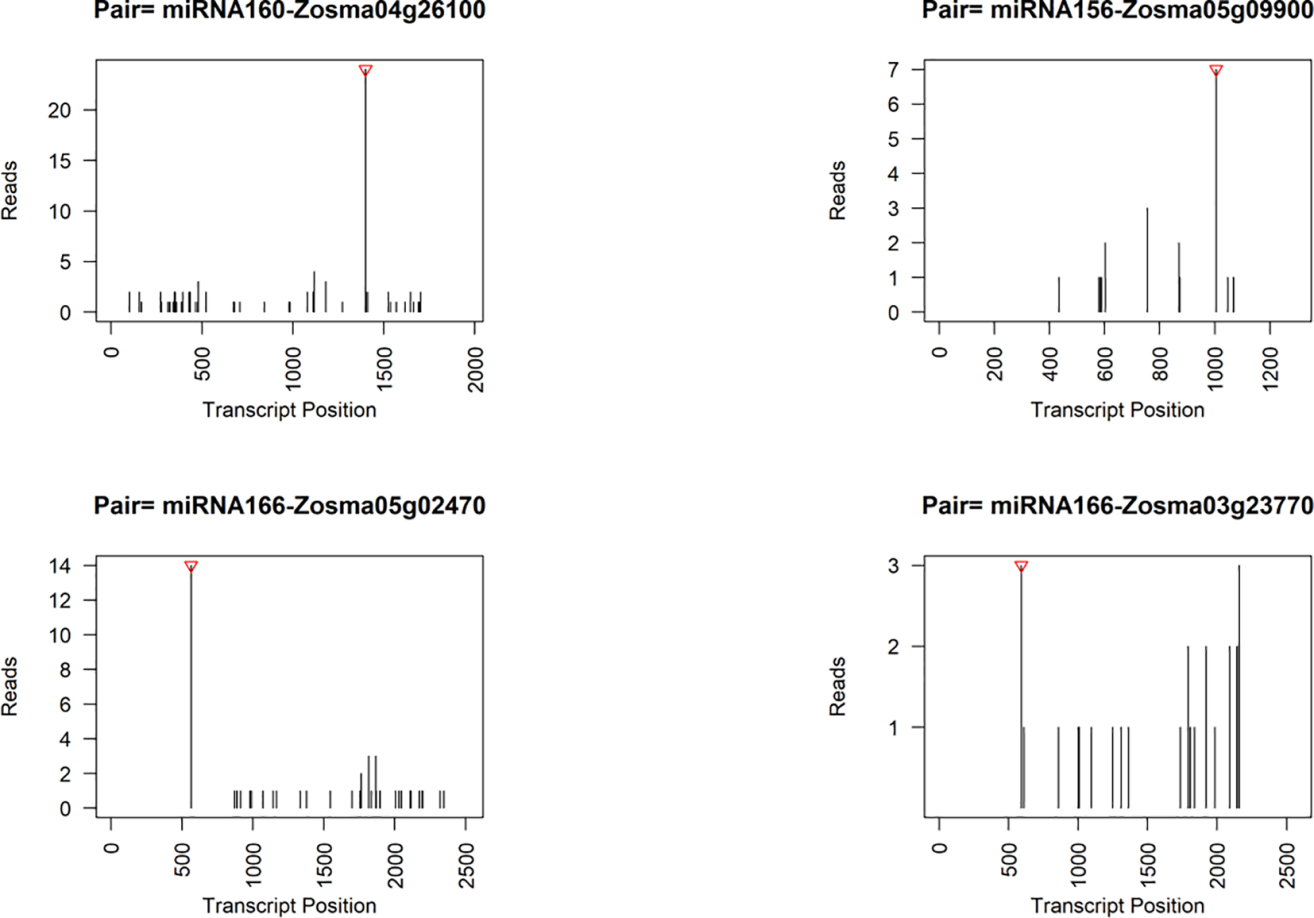
Representative target plots (t-plot) depicting categories of the cleaved mRNAs confirmed by degradome sequencing. The red triangle at the top represents the predicted cleavage location.

We also employed degradome sequencing to investigate the target sites of microRNAs (miRNAs), resulting in identification of 91 reliable miRNA-target pairs (**Supplementary Data S8**). 86.8% of these miRNA-target pairs were consistent with the predicted pairs identified by psRNATarget. Among the predicted targets, we found two genes were DEGs in response to salt stress. *Zosma04g26100*, a potential target of miRNA160 (**Figure 6**), exhibited up-regulation in SR tissue. The homolog of *Zosma04g26100*, *athARF16* (*AT4G30080*) plays roles in root cap cell differentiation and is regulated by both miRNA160 and plant hormones (Wang et al., 2005; Bascom, 2023). The relatively insignificant response of miRNA160 might facilitate the increased expression of *Zosma04g26100*. *Zosma05g29470*, identified as a potential target for NOVEL056, displayed a predicted cutting site that is not located near the peak of degraded fragments (**Supplementary Figure S5**). The homologous gene *athPAL1* (*AT2G37040*) of *Zosma05g29470* is implicated in the biosynthesis of lignin and flavonoid (Olsen et al., 2008), suggesting that the down-regulation of *Zosma05g29470* could adversely affect related metabolic functions.

Although some genes did not exhibit obvious responses to salt stress, they were potentially regulated by microRNAs, as indicated by the degradome data and previous studies (**Figure 6**). For instance, the target gene of miRNA156, *Zosma05g09900*, is a member of the SPL family, of which homologous gene has been implicated in various processes such as plant morphogenesis (Ferreira e Silva et al., 2014), secondary metabolism (Gou et al., 2011), and signal transduction (Barrera-Rojas et al., 2023). *Zosma05g02470* and *Zosma03g23770*, both belonging to the HD-ZIP family (**Supplementary Figure S5**), are predicted target genes of miR166. Their homologous genes in *A. thaliana* play crucial roles in regulating vascular development (Kim et al., 2005). Moreover, the degradome data provided evidence for additional miRNA-target pairs, including miR396-GRF (*Zosma02g02440*/*Zosma01g12870*), miR393-TIR (*Zosma01g13700*/*Zosma02g25510*), miR167-ARF (*Zosma01g35720*), miR319-TCP (*Zosma03g04610*), and miR164-NAC (*Zosma05g19590*) (**Supplementary Figure S5**), which have been reported to function in other plant species (Guo et al., 2005; Gutierrez et al., 2009; Liang et al., 2014; Koyama, 2017; Wang et al., 2018). The miRNA cleavage sites identified by the degradome are speculative and require future verification.

## 4 Discussion

### 4.1 Osmosis homeostasis at high salinity

The enriched GO-term “response to water deprivation” suggests that eelgrass may experience osmotic stress similar to drought conditions in high salt environment. Most DEGs within this GO-term are significantly up-regulated in leaf, including *Zosma01g16060*, *Zosma03g14850*, *Zosma03g23560, Zosma03g33910*, *Zosma03g37150*, *Zosma05g03710* and *Zosma05g33490*. Among these genes, *Zosma01g16060* is a member of the PP2C family, of which homolog in *A. thaliana*, *HAI2* (*AT1G07430*), whose mutant has been documented to enhance drought resistance by regulating proline and osmoregulatory solute accumulation (Bhaskara et al., 2012). The other two DEGs *Zosma05g03710* and *Zosma03g14850* in GO term of “response to water deprivation” also appear to be involved in salt stress. The homolog (*AT1G01470*) of *Zosma05g03710* has been reported to exhibit significant protective effects under salt stress (Jia et al., 2014), while the homolog *RCI2B* (*AT3G05890*) of *Zosma03g14850* is responsive to abiotic stresses such as low temperature and water scarcity, with its expression induced by ABA (Capel et al., 1997). *Zosma05g33490* and *Zosma03g37150* belong to the *AP2/ERF* family. The homologous gene *DREB1A* (*AT4G25480*) of *Zosma05g33490* has been implicated in the response to abiotic stresses, including drought, across various plant species (Polizel et al., 2011; Ravikumar et al., 2014). Meanwhile, the homolog of *Zosma03g37150* is known to participate in gene expression related to dehydration and cold stress (Sakuma et al., 2002). Besides, the collinear genes of *Zosma01g16060*, *Zosma05g33490*, and *Zosma05g03710* in rice and maize also exhibit responses to salt stress, suggesting a conserved role among these “response to water deprivation” genes (**Supplementary Data S10**; **Supplementary Figure S6**). By comparing the enriched go-terms, we observed that the biological process “response to water deprivation” is conserved in three species (**Supplementary Figure S6C**). Within this go-term, some gene families, such as AP2, PP2C, and Annexin, are present in both eelgrass and rice, suggesting similarities in how marine and terrestrial plants respond to high salinity stress.

Aquaporins are the key regulators for regulating osmotic homeostasis in plants (Ren et al., 2021). Previous studies in other seagrass have suggested that aquaporins contribute to the adaptation in hypersaline environments (Sandoval-Gil et al., 2023), and one of the studies has shown that aquaporins are involved in the maintenance of osmotic balance (Serra et al., 2013). In this study, we identified a total of 25 aquaporins in eelgrass (**Supplementary Data S11**). The gene expression profile revealed that several aquaporin members, including *Zosma01g17610*, *Zosma01g27210*, *Zosma02g03080*, *Zosma02g23130*, and *Zosma03g25250*, exhibited high expression levels across multiple organs (**Supplementary Figure S7A**). The phylogenetic analysis (**Supplementary Figure S7B**) indicates that *Zosma01g17610* belongs to the TIP branch, while *Zosma01g27210*, *Zosma02g23130*, and *Zosma02g03080* belong to the SIP branch, and *Zosma03g25250* is part of the NIP branch. Members of the TIP branch have been associated with the promotion of new lateral root primordia (Reinhardt et al., 2016), whereas SIP branch members are primarily localized in the endoplasmic reticulum, functioning as channels for water, small molecules, and ions (Ishikawa et al., 2005). Additionally, NIP branch members are implicated in the absorption of certain mineral ions and can influence hydrogen peroxide (H_2_O_2_) levels (Kamiya et al., 2009; Kamiya and Fujiwara, 2009; Wang et al., 2017). The widespread expression of these genes may facilitate the transport of water and small solutes across cell membranes in eelgrass. Simultaneously, we observed that three aquaporins (*Zosma03g21300*, *Zosma01g24240*, and *Zosma03g27440*) exhibited significantly down-regulated expression levels under high salt conditions. The phylogenetic tree reveals that *Zosma01g24240* belongs to the PIP1 branch, *Zosma03g21300* to the PIP2 branch, and *Zosma03g27440* to the TIP branch. Members of the PIP1 branch are reported to be involved in water transport and immune responses (Postaire et al., 2010; Tian et al., 2016), while those in the PIP2 branch are associated with drought resistance (Chen et al., 2021, 2022) and the transport of H_2_O_2_ (Rodrigues et al., 2017; Wang et al., 2020). *Zosma03g27440* and *Zosma01g17610* share the same branch, suggesting potential functional redundancy. Furthermore, a number of their homologous genes in rice and maize, such as *Zm00001eb096680*, also exhibit reduced expression at high salinity (**Supplementary Figure S7C**). In terrestrial plants, drought or salt stress can suppress the expression of certain aquaporins such as PIPs, thereby reducing root hydraulic conductivity by limiting water loss (Yepes-Molina et al., 2020). The down-regulation of aquaporins in eelgrass might reflect a conserved character in plants responding to salt stress.

### 4.2 Ion homeostasis at high salinity

Plant adaptation to high-salinity environments depends in part on the maintenance of cell ion homeostasis, a process significantly facilitated by ion transporters (Malakar and Chattopadhyay, 2021). In this study, we identified several key ion transporter families in eelgrass, including five Na(+)/H(+) exchangers (NHX), five K(+) efflux antiporters (KEA), fifteen cation/H(+) exchangers (CHX), and eleven sodium/calcium exchanger proteins belonging to CAX family (**Supplementary Data S11**). The gene expression profile (**Supplementary Figure S8A**) indicates that most NHX and KEA genes are broadly expressed across multiple organs, with the exception of one NHX member (*Zosma01g13710*), which exhibits specific expression in floral organs. In contrast, the expression of CHX family exhibits organ specificity, particularly with higher expression levels in male flowers, suggesting a potential role in reproductive development. Approximately half of the CAX members are also widely expressed in various organs, including *Zosma02g20240*, which shows significant accumulation under high salt stress. While alterations in calcium levels are critical in the SOS pathway (Ali et al., 2023), there is currently no evidence linking CAX family members to this process. Previous research has indicated that certain CAX family members, such as *athNCL* (*AT1G53210*), contribute to plant salt tolerance (Wang et al., 2012). The genes of eelgrass in the same branch of *athNCL* include *Zosma01g22450* and *Zosma06g26160* (**Supplementary Figure S8B**), while *Zosma02g20240* is positioned on a separate branch, indicating the need for further investigation into their roles in salt tolerance. Additionally, comparative analysis reveals that, unlike eelgrass, many ion channel protein genes in rice and maize respond to salt stress (**Supplementary Figure S8C**), highlighting the differences in ion regulation mechanisms between terrestrial and marine plants.

### 4.3 Activation of antioxidant system and secondary metabolic pathways

Osmotic stress and ionic stress induced by salt stress can lead to the production of ROS in plants (Hasanuzzaman et al., 2021). To mitigate the effects of ROS, plants can accumulate osmotic products and enhance the activity of antioxidant enzymes, thereby improving their tolerance to salt stress (Yang and Guo, 2018). In this study, several antioxidant enzymes were identified in eelgrass, including SOD, CAT, GPX, and APX (**Supplementary Data S11**). Gene expression profiles revealed that, while most antioxidant enzymes did not show a significant response to salt stress, they were highly expressed across multiple organs (**Supplementary Figure S9A**). Zhang et al. (2023) also discovered the upregulation of SOD, peroxidase and other antioxidant enzyme genes in germination of eelgrass, but with no significant difference. Olsen et al. (2016) reported many stress-resistance genes in eelgrass. The salinity and temperature fluctuation are usually gradual in coastal environments, we speculate that high constitutive expression of antioxidant enzyme genes in eelgrass might be the adaptation mechanism to reduce the ROS during the gradual change of abiotic stress. Notably, *Zosma06g29150*, a member of the GPX family, exhibited significant up-regulation in the leaf, indicating that this enzyme may function in reducing H_2_O_2_ by oxidizing glutathione (GSH) (Das and Roychoudhury, 2014), thereby enhancing salt tolerance. GPX was also reported as the most abundant DEGs and proteins in eelgrass under low salinity stimulation during germination (Zhang Y. et al., 2023), which is coincident with the result of our study. It indicated that GPX family is the main antioxidant enzyme for scavenging ROS in eelgrass. Furthermore, homologous genes of *Zosma41g01010* in rice and maize, such as *Os02g0664000* and *Zm00001eb016270*, also respond to salt stress (**Supplementary Figures S9B, C**).

Salt stress induces secondary metabolic changes in plants, especially the accumulation of phenolic compounds, including lignin (Dabravolski and Isayenkov, 2023) and flavonoids (Shomali et al., 2022). Lignin fortifies plant cell walls, thereby protecting membrane integrity under salt stress. Flavonoids play a role in reducing ROS accumulation to enhance plant resistance to adverse conditions (Li et al., 2018). The biosynthesis of many phenolic compounds occurs via the phenylpropanoid pathway (Salam et al., 2023). Transcriptional analysis in this study revealed that several genes associated with phenylpropanoid and flavonoid synthesis were responsive to high salt stress. This suggests that the synthesis of phenolic compounds in eelgrass is highly active under such conditions. Furthermore, homologous genes in rice and maize exhibited significant up-regulation (**Supplementary Figure S10**), supporting the conserved secondary metabolic responses to salt stress across plant species.

### 4.4 Activation of phytohormone signaling pathway

Plant hormones are important participants in the salt tolerance of plants (Yu et al., 2020). In eelgrass, we identified a series of hormone-related genes responding to salt stress, suggesting high salinity stimulates the activation of phytohormone signaling pathway.

We have mentioned in the result that the ABA synthesis gene, *Zosma04g22590*, a member of the NCED family, was consistently up-regulated across three comparisons. However, another NCED family member, *Zosma01g20220*, exhibited down-regulation. In rice and maize, most members of this family are up-regulated (**Supplementary Figure S11**), suggesting the conservation of ABA signaling in plant salt responses. This viewpoint is further supported by several potential downstream genes of the ABA signaling pathway, including dehydrin family protein (*Zosma03g27730*), protein kinases (*Zosma03g25920*), and PP2C family members (*Zosma01g41250*, *Zosma02g21670*). Their expressions were up-regulated at high salinity and homologous genes have been reported to be induced by ABA (**Supplementary Figure S11**). *RAB18* is the homolog of *Zosma03g27730* and reported to interact with *PIP2B* (Hernández-Sánchez et al., 2019), which suggests the regulation of aquaporin genes by ABA signaling. *Zosma01g41250* and *Zosma02g21670* are members of the PP2C family. Phylogenetic tree analysis shows that *Zosma01g41250* is located on the branch with *HAI1* and *HAI2*, while *Zosma02g21670* is located on the branch with *PP2C5* and *AP2C1*(**Supplementary Figure S11**). It is reported that HAI PP2C mutants had enhanced proline and osmoregulatory solute accumulation at low water potential (Bhaskara et al., 2012). Both *PP2C5* and *AP2C1* function as MAPK phosphatases, which are important to ABA signaling (Brock et al., 2010; Schweighofer et al., 2007). The up-regulation of PP2C members seems to be a conserved character under salt stress, as many homologous genes in rice and maize also show similar responses (**Supplementary Figure S11**). Terrestrial plants typically respond to osmotic stress caused by high salinity by regulating stomatal opening and closing via ABA (Hedrich and Shabala, 2018; Komatsu, 2020). Since eelgrass lacks stomata (Olsen et al., 2016), how ABA helps eelgrass cope with high-salinity environments needs further investigation.

In addition, JA, auxin, brassinosteroid (BR), cytokinin (CK) and gibberellin (GA) are also potential regulatory factors in the salt stress response of eelgrass. Research indicates that JA signaling in terrestrial plants is activated in response to salt stress, thereby enhancing salinity tolerance (Valenzuela et al., 2016; Zhao et al., 2014). Among the DEGs identified in eelgrass, *Zosma01g30620*, *Zosma05g15520*, and *Zosma06g28020* are associated with JA signaling and down-regulated at high salinity. The homologous gene of *Zosma01g30620*, lipoxygenases (LOX3) is involved in JA synthesis (López-Cruz et al., 2014). The homolog of *Zosma05g15520*, jasmonic acid carboxyl methyltransferase (JMT) is a carboxyl methyltransferase that catalyzes the formation of methyl jasmonate from jasmonate (Seo et al., 2001). *Zosma06g28020* is homologous to the jasmonic acid receptor *COI1* (Song et al., 2021). Furthermore, we found that auxin-related genes *Zosma03g17780* and *Zosma05g12790* were down-regulated under high salt stress. The homolog of *Zosma03g17780*, *PGP4* encodes an auxin effector transmembrane transporter, which is involved in auxin transport and inhibits root hair elongation (Cho et al., 2007). The homolog of *Zosma05g12790*, *ARA-2* plays a role in the auxin signaling pathway and inhibits lateral root development (Koh et al., 2009). Exogenous application of BR can enhance the salt tolerance of terrestrial plants, indicating that BR may be involved in salt tolerance (Fu and Yang, 2023). *Zosma03g35130* and *Zosma03g30090* are identified as BR-related genes. Overexpression of the *BKI1*, a homolog of *Zosma03g35130*, leads to a salt-tolerant phenotype (Zhang et al., 2017). *DOGT1*, a homolog of *Zosma03g30090*, encodes a don-glucosyltransferase that regulates BR activity (Poppenberger et al., 2005). Moreover, *Zosma06g28530* and *Zosma01g36380*, family members of two-component response regulator *ARR-A*, are associated with CK (Li et al., 2013; Ishida et al., 2008). These two genes are down-regulated at high salinity in eelgrass, most of which homologous genes in maize exhibited similar responses (**Supplementary Figure S11**). Among the down-regulated DEGs in SR tissue, we identified one gene related to gibberellin synthesis, *Zosma06g07970* (ent-kaurene oxidase), suggesting that hypersalinity may regulate gibberellin levels in eelgrass.

In terrestrial plants, the actions of different phytohormones are interconnected. For instance, the key regulator jasmonate ZIM-domain proteins (JAZ) in JA could be regulated by the PYL6-MYC2 module of ABA (Aleman et al., 2016). CK receptor kinase significantly regulates ABA levels (Nishiyama et al., 2013). The interplay between ABA and GA signaling under abiotic stress conditions is significantly influenced by DELLA proteins (Aizaz et al., 2024). In the future, we aim to reveal the reasons for these transcriptional changes by studying the hormone content in eelgrass. Furthermore, exploring the potential interactions of phytohormones under salt stress in eelgrass will be valuable.

### 4.5 SOS and MAPK signaling pathway in eelgrass

The SOS pathway is crucial for plant salt tolerance. Its core components include SOS1 (sodium-proton exchanger), SOS2 (CBL-interacting protein kinase), SOS3 (calcium-binding EF-hand family protein), and SCaBP8 (SOS3-like calcium-binding protein). Additional components associated with SOS pathway, such as *HIS1-3*, *MPK4*, *PLATZ2*, *RSA1* at the transcriptional level, and *UBC*, as well as *14-3-3*, *BIN2*, *CIPK8*, *GI*, *PKS5*, and *PP2C* at the post-transcriptional level, were also identified for plant salt tolerance (Ali et al., 2023). In our study, we utilized OrthoFinder (**Supplementary Data S11**) to identify SOS pathway genes in eelgrass. We found that none of these genes exhibited a significant response to salt stress, which was different from the findings in rice and maize (**Supplementary Figure S12**). Unlike terrestrial plants, which only have their roots in direct contact with soil salinity change, the whole plant of seagrass is exposed to the hypersalinity. The SOS pathway genes show high expression levels in various organs in eelgrass (**Supplementary Figure S12A**), which may provide a foundation for the protein-level responses of SOS pathway. Furthermore, several genes including *Zosma01g22480* (*SCaBP*), *Zosma05g05710* (*14-3-3*), and *Zosma01g41780* (*BIN2*), were specifically expressed in floral organs, indicating their potential involvement in the reproductive development of eelgrass.

Salt stress can activate the MAPK signaling cascade, which involves protein kinases that phosphorylate various substrates (Taj et al., 2010). In this study, we observed that the expression of two MAPKKK genes, *Zosma03g00830* and *Zosma03g00810*, was down-regulated under high salt conditions in leaf. Their *A. thaliana* homolog *MAPKKK18* (*AT1G05100*) is induced by ABA and promotes leaf senescence (Matsuoka et al., 2015). This suggests that eelgrass may enhance its salinity tolerance by reducing the accumulation of these MAPKKK genes, thereby avoiding senescence. Additionally, the colinear gene *Os05g0545300*, corresponding to *Zosma03g00810*, also responded to salt stress, although it exhibited down-regulation in only one comparison (**Supplementary Figure S13**).

### 4.6 miRNA and their potential targets in eelgrass

We identified a total of 172 MIRNA loci using small RNA-seq data. The mature sequences derived from these MIRNAs were predominantly 21 nucleotides in length. 83 (71.6%) of these microRNAs had uracil (U) as the first base at the 5’ end. Previous studies have demonstrated that the presence of uracil at the 5’ end can enhance the binding of microRNAs to the AGO1 protein (Mi et al., 2008). Based on the similarity of mature miRNAs, we identified 38 MIRNA belonging to known families, which is far fewer than the 93 known conserved miRNAs predicted genome-wide by Ma et al. (2021). The limited number of known miRNAs might be attributed to the restrictions in the plant tissues or RNA extraction method. The same kits for total RNA extraction were used for both mRNA and small RNA sequencing, as they have been employed in other researches (Zhang M. et al., 2023; Hu et al., 2021). In order to prove the accuracy of our study, we have experimentally validated the response of some DEmiRNAs using qRT-PCR. The results proved the existence of those miRNAs and the expression pattern of them was the same as the result from library construction.

Differential expression analysis of miRNAs revealed multiple miRNAs response to salt stress, including miR156, miR166, miR172, and a number of *de novo* miRNAs. miR156 has been reported to participate in salt tolerance across different plant species such as apple (Ma Y. et al., 2021), tobacco (Kang et al., 2019) and Alfalfa (Arshad et al., 2017). Similarly, miR166 has been shown to respond to salt stress in plants such as *Hemerocallis fulva* (Zhou et al., 2023), sugar beet (Zhang Z. et al., 2023), and potato (Kitazumi et al., 2015). It also plays a regulatory role in other abiotic stresses, including drought (Zhao et al., 2024) and low potassium conditions (Lei et al., 2023). In cereal crops, miR172 contributes to salt tolerance by maintaining ROS homeostasis (Cheng et al., 2021). Based on expression correlation analysis, we hypothesize that miR156 and miR166 are key regulators of the salt stress response in eelgrass. Specifically, miR156 was significantly up-regulated in leaf, while three of its target genes, WRKY family member *Zosma06g26970*, cellulose synthase *Zosma04g03580*, and heavy metal-related domain protein *Zosma04g09250*, were significantly down-regulated. Similarly, miR166 exhibited significant up-regulation in leaf, with two of its target genes, HD-ZIP family member *Zosma03g00210* and S-locus lectin protein kinase family protein *Zosma04g15070*, also showing significant down-regulation. This negative correlation in expression patterns suggests the potential existence of miRNA-target modules involved in the salt stress response. Meanwhile, the degradome data predicted additional miRNA-target pairs (**Figure 5**; **Supplementary Figure S5**), which require further verification of their function in eelgrass.

Several microRNAs, including miR160 (Tang et al., 2022), miR164 (Lu et al., 2017), miR167 (Ye et al., 2020), miR172 (Cheng et al., 2021), miR319 (Zhou et al., 2013), miR390 (Chu et al., 2022), miR393 (Denver and Ullah, 2019), miR396 (Pegler et al., 2021), miR398 (He et al., 2021), miR399 (Pegler et al., 2020), and miR528 (Wang et al., 2021), have been reported to respond to salt stress or contribute to salt tolerance in various plant species. Although these miRNAs were identified in eelgrass, their expression levels did not exhibit significant differences under salt stress. However, some target genes of these miRNAs are responsive to salt stress (**Supplementary Data S7**), such as *Zosma01g26740*, a predictive target gene for miR167, and *Zosma03g30130*, a predictive target gene for miR319. *Zosma01g26740* belongs to the *ARF* family and shows significantly reduced transcription levels under salt stress. *ARF8* (*AT5G37020*), which is homologous to *Zosma01g26740*, has been reported to be regulated by miR167 and involved in root development (Gutierrez et al., 2009; Liao et al., 2023). Similarly, *Zosma03g30130*, a member of MYB family, exhibits down-regulated transcription levels under salt stress. *MYB33* (*AT5G06100*), which is homologous to *Zosma03g30130*, is reported to be regulated by miR319 and specifically down-regulated in roots treated with ethylene, thereby influencing root growth (Zhang et al., 2016). The interaction between these prediction targets and miRNAs is worthy of further exploration in eelgrass.

### 4.7 Essential roles of TFs in the response to salt stress

The results revealed that 81% of salt-responsive TFs were distributed in eight modules: M1, M2, M3, M4, M5, M6, M7, and M9. A homologous analysis of these TFs indicated that several homologous genes from the bHLH, ERF, and MYB families in rice and maize exhibited similar expression patterns under high salt stress (**Supplementary Figure S14**). For instance, the bHLH family member *Zosma03g02210*, *Os04g0300600*, *Os04g0301500*, and *Zm00001eb086370* are grouped in a branch. Similarly, ERF family members *Zosma03g37150* and *Os06g0166400* are found in the same branch, while ERF family members *Zosma02g05080* and *Os12g0168100* are collinear genes in one branch. Additionally, MYB family members *Zosma06g17610*, *Zosma06g17660*, *Os03g0720800*, *Os11g0207600*, and *Zm00001eb168720* are grouped together in a branch. The consistent up-regulation of these genes suggests that they may function as conserved TFs involved in response to high salinity.

We focused on TFs that potentially regulate enriched biological processes and pathways, including GO:0009414 (response to water deprivation), map04016 (MAPK signaling pathway - plant), map00940 (phenylpropanoid biosynthesis), and map01110 (biosynthesis of secondary metabolites). To evaluate the expression correlation between DEGs, we calculated the Pearson correlation coefficient, considering values of ≥0.8 as indicative of similarity. The results revealed the potential regulatory roles of several TFs (**Supplementary Figure S15**). For instance, *Zosma04g22590* (NCED) and *Zosma02g25150* (arogenate/prephenate dehydratase) may be targeted by the ERF family member *Zosma05g04820*. *Zosma03g22300* (phenylalanine ammonia-lyase) may be regulated by MYB family members *Zosma06g17610* and *Zosma06g17660*. Furthermore, numerous potential binding sites for TFs are located in the upstream regions of microRNAs that respond to salt stress (**Supplementary Figure S4**). Further research is needed to determine whether these TFs can effectively regulate microRNA expression.

### 4.8 Summary

In this study, we performed a comprehensive analysis to reveal the response of eelgrass to high salt using transcriptome and degradome, and compared the result with other transcriptomes obtained from NCBI. In total, 14 DEmiRNAs and 691 DEGs including 53 TFs were identified. We further predicted the interactions between TFs, miRNAs, and their potential target genes. For instance, the up-regulated ERF members in the leaf may function by regulating the ABA synthase NCED, while MYB members might affect secondary metabolism by regulating PAL. Additionally, miRNA156-WRKY and miRNA166-HD-ZIP are supposed to be regulatory modules influencing the response on the post-transcriptional level.

Through the identification of homologous genes and expression profiling, we obtained a series of conserved responsive genes in eelgrass and two terrestrial plants rice and maize. The osmotic response related to the go-term “response to water deprivation” seems to be conserved in three species. Besides, we identified other conserved responses between rice and maize (**Supplementary Figure S16**), which suggest potential differences between terrestrial plants and marine plants in response to salt stress, such as the “gibberellin biosynthetic process”. Moreover, eelgrass exhibits specific responses to high salinity, as evidenced by the enriched go-terms such as fucose biosynthesis and vacuolar protein processing. These findings enhance our understanding of the molecular-level adaptability of seagrass to marine environments. In the future, we aim to validate the functions of these genes and their homologs in model organisms through experiments such as constructing overexpression plants. The conserved and specific responsive genes can serve as potential genetic resources for improving the salt tolerance of crops.

## Data Availability

The RNA-seq, small RNA-seq and degradome data used in this study has been deposited into CNCB database (https://www.cncb.ac.cn/) with the accession number of CRA020957 (BioProject: PRJCA031998). The other datasets for detailed analysis were provided in supporting information files.
