# Supplementary material for: New insights into the salt-responsive regulation in eelgrass at transcriptional and post-transcriptional levels
Source: Front Plant Sci. 2025 Feb 6;16:1497064. doi: 10.3389/fpls.2025.1497064 (PMC11840677; doi:10.3389/fpls.2025.1497064)

Supplementary Material

1 Supplementary Data

The supplementary data sheets are available in Supplementary_Data.xlsx, including:

Supplementary Data S1

The differentially expressed genes（DEGs）identified in leaf and the mix tissue of stem and root

Supplementary Data S2

The sra accession obtained from NCBI used for WCGNA co-expression network construction.

Supplementary Data S3

The list of genes in WGCNA modules.

Supplementary Data S4

The list of transcription factors identified by plantTFDB.

Supplementary Data S5

The microRNAs of eelgrass identified by miRDeep-P2 and ShortStack.

Supplementary Data S6

The DEmiRNAs identified by edgeR and DEseq2.

Supplementary Data S7

The potential target genes of microRNAs identified by psRNATarget.

Supplementary Data S8

miRNA-mediated cleavage of target mRNA identifed by degradome sequencing.

Supplementary Data S9

The sra accession obtained from NCBI used for comparative transcriptome analysis.

Supplementary Data S10

The collinear genes in eelgrass and another 3 species.

Supplementary Data S11

The identified genes in specific gene families mentioned in discussion.

Supplementary Data S12

The primers used for qRT-PCR.

2 Supplementary Figures

Supplementary Figure S1

A. The number of DEGs in response to salt stress. The red part of bars represent up-regulated genes, while the blue part of bars represent down-regulated genes. “LF” refers to the leaf; “SR” refers to the mix tissue of stem and root.

B. The Venn diagram which shows 3 sets of DEGs. “Rg” refers to all responsive genes. “Up” refers to the up-regulated genes. “Dn” refers to the down-regulated genes.

C. Enriched Mapman items in response to salt stress in leaf.

D. Enriched Mapman items in response to salt stress in mix tissue of stem and root.

E. Enriched GO-terms of tissue-specific gene sets in response to salt stress.

Supplementary Figure S2

A. Scale-free topology model Fit and mean connectivity of WCGNA network, which reveal the softpower chosen for WCGNA network construction is 8.

B. The expression pattern of module Eigengenes of WCGNA network.

C. The number of DEGs in each module of WCGNA network.

D. The response of DEGs in M4, M6 and M9 mentioned in the second paragraph of 3.2.

E. The number of responsive TFs in each module of WCGNA network.

F. The number of DEGs with cis-regulatory elements on the upstream region, which are predicted by FIMO and potentially bound by TFs.

G. The number of DEGs with cis-regulatory elements on the upstream region, which are predicted by plantCARE.

Supplementary Figure S3

A. Genomic distribution of MIRNAs.

B. The number of intergenic and intragenic MIRNAs.

C. The position of intragenic MIRNA within the gene.

D. The histogram which shows the distance of intergenic MIRNA from upstream gene.

E. Venn diagram of DEmiRNAs identified by edgeR and DESeq2.

F. The expression of three additional up-regulated DEmiRNAs was validated using qRT-PCR.

Supplementary Figure S4

A. The number of MIRNAs with cis-regulatory elements on the upstream region, which are predicted by FIMO and potentially bound by TFs.

B. The number of DEmiRNAs with cis-regulatory elements on the upstream region, which are predicted by FIMO and potentially bound by TFs.

C. The number of DEmiRNAs with cis-regulatory element on the upstream region, which are predicted by plantCARE.

Supplementary Figure S5

Representative target plots (t-plot) depicting categories of the cleaved mRNAs confirmed by degradome sequencing. The red triangle at the top represents the predicted cleavage location.

Supplementary Figure S6

Homologous gene analysis of the DEGs involved in water deprivation in eelgrass.

A. Phylogenetic tree of a particular gene and its top 5 homologous genes.

B. The response to salt stress of homologous genes in rice or maize of particular genes in eelgrass. The homologous genes and corresponding genes were separated by "|".

C. The overlap of enriched GO-terms identified from up-regulated genes of eelgrass (leaf), rice and maize in response to salt stress.

Supplementary Figure S7

Expression and homologous gene analysis of aquaporin.

A. Expression profile of aquaporin in eelgrass.

B. Phylogenetic tree of aquaporin in eelgrass and other 3 species.

C. The response to salt stress of aquaporin in rice or maize.

Supplementary Figure S8

Expression and homologous gene analysis of ion transporters in eelgrass.

A. Expression profile of ion transporters (NHX, KEA, CHX and CAX) in eelgrass.

B. Phylogenetic tree of ion transporters in eelgrass and their top 5 homologous genes in other 3 species.

C. The response to salt stress of ion transporters (top 5 homologous genes to the ones in eelgrass) in rice or maize. The homologous genes and corresponding class were separated by “|”.

Supplementary Figure S9

Expression and homologous gene analysis of antioxidant enzymes in eelgrass.

A. Expression profile of antioxidant enzymes (SOD, CAT, GPX and AXP) in eelgrass.

B. Phylogenetic tree of antioxidant enzymes in eelgrass and their top 5 homologous genes in other 3 species.

C. The response to salt stress of antioxidant enzymes (top 5 homologous genes to the ones in eelgrass) in rice or maize. The homologous genes and corresponding class were separated by “|”.

Supplementary Figure S10

Homologous gene analysis of the DEGs involved in secondary metabolites in eelgrass.

A. Phylogenetic tree of a particular gene and its top 5 homologous genes.

B. The response to salt stress of homologous genes in rice or maize of particular genes in eelgrass. The homologous genes and corresponding genes were separated by “|”.

Supplementary Figure S11

Homologous gene analysis of the DEGs associated with phytohormone in eelgrass.

A. Phylogenetic tree of particular genes and their top 5 homologous genes.

B. The response to salt stress of homologous genes in rice or maize of particular genes in eelgrass. The homologous genes and corresponding gene were separated by “|”.

C. Homologous gene list of DEGs in eelgrass related to phytohormones.

Supplementary Figure S12

Expression and homologous gene analysis of genes in SOS signal pathway.

A. Expression profile of genes in SOS signal pathway in eelgrass.

B. The response to salt stress of genes in SOS signal pathway (identified by orthoFinder) in rice or maize.

Supplementary Figure S13

Homologous gene analysis of the DEGs involved in MAPK signaling pathway in eelgrass.

A. Phylogenetic tree of particular genes and their top 5 homologous genes.

B. The response to salt stress of homologous genes in rice or maize of particular genes in eelgrass. The homologous genes and corresponding gene family were separated by "|".

Supplementary Figure S14

Expression and homologous gene analysis of TFs in eelgrass.

A. Phylogenetic tree of differential expressed TFs in bHLH, ERF, MYB family in eelgrass and their top 5 homologous genes in other 3 species.

B. The response to salt stress of three family TFs (top 5 homologous genes to the ones in eelgrass) in rice or maize. The homologous genes and corresponding gene family were separated by "|".

Supplementary Figure S15

Network of DEGs in enriched bioprocess or pathway potentially regulated by responsive TFs under hypersaline environment.

Supplementary Figure S16

The overlapping enriched GO-terms identified from up-regulated genes of rice and maize in response to salt stress.

Supplementary Figure S1

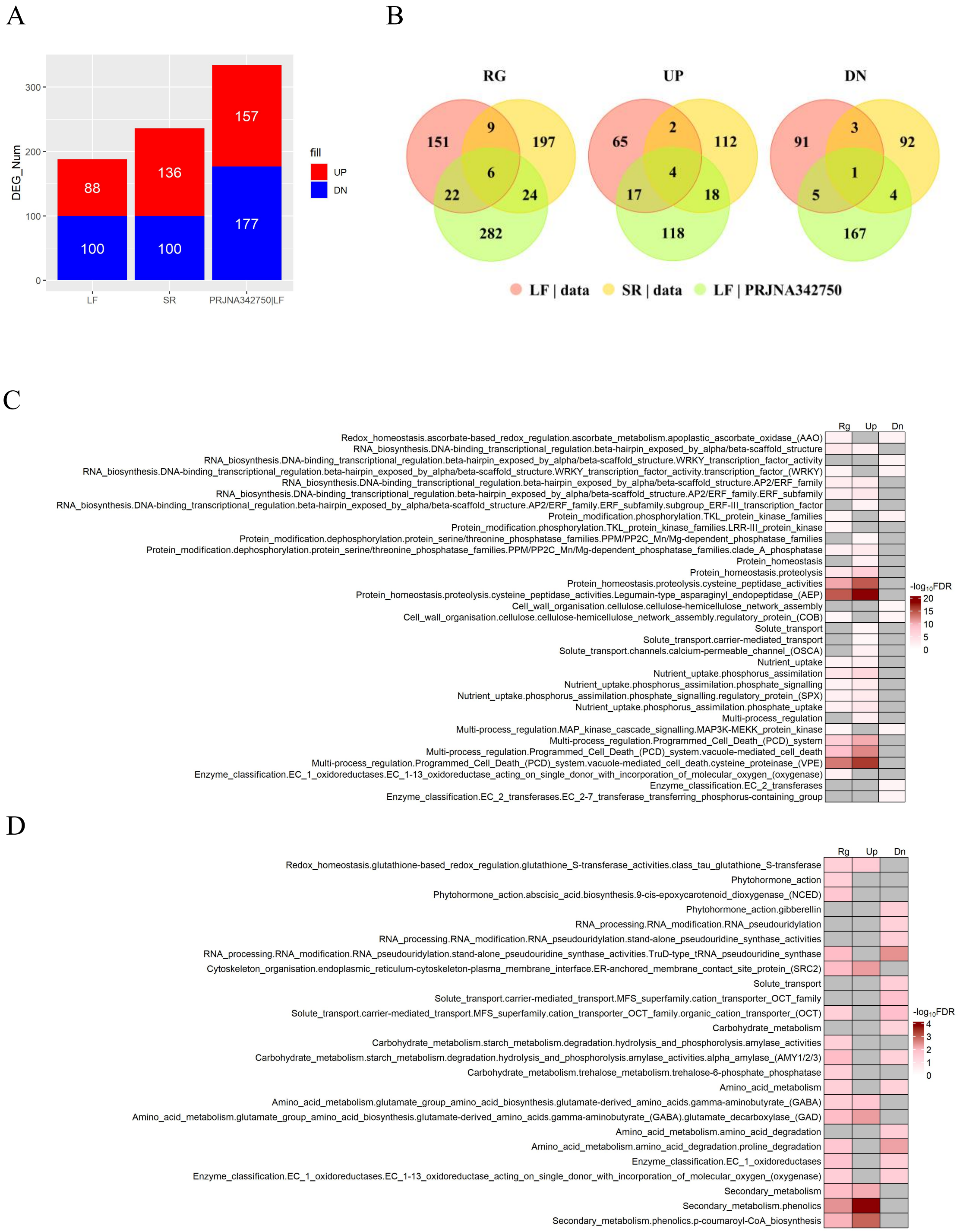

E

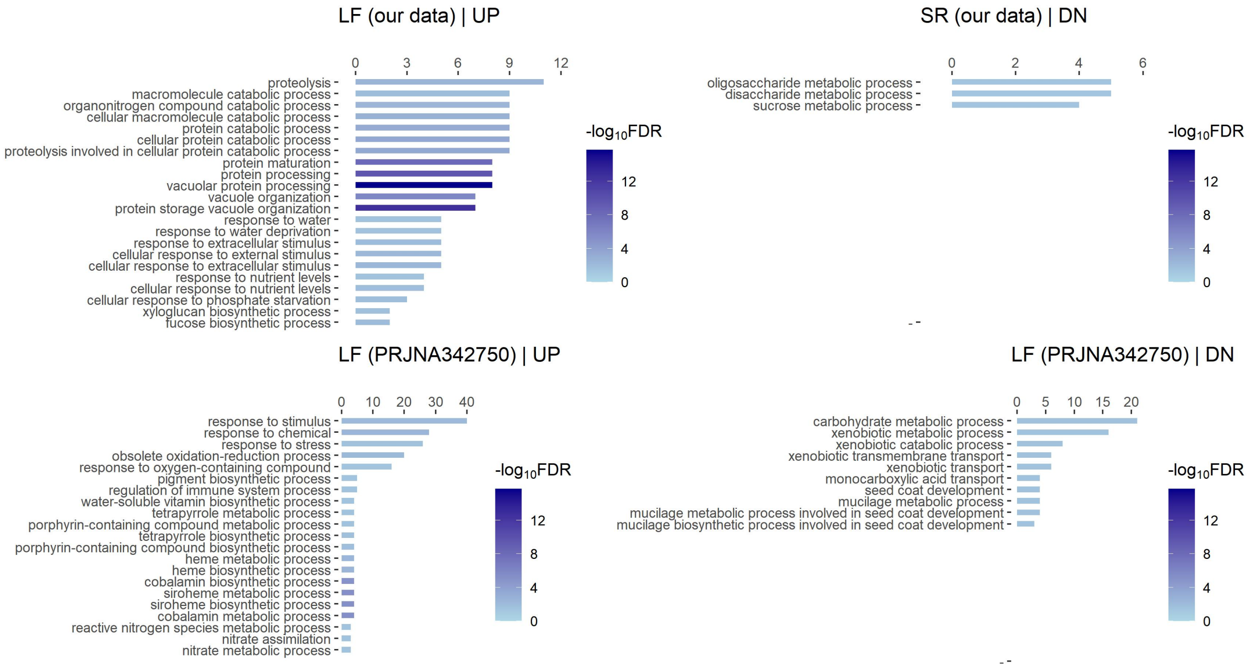

LF (our data) | UP
0
3
6
9
12
proteolysis
macromolecule catabolic process
organonitrogen compound catabolic process
cellular macromolecule catabolic process
protein catabolic process
cellular protein catabolic process
proteolysis involved in cellular protein catabolic process
protein maturation
protein processing
vacuolar protein processing
vacuole organization
protein storage vacuole organization
response to water
response to water deprivation
response to extracellular stimulus
cellular response to external stimulus
cellular response to extracellular stimulus
response to nutrient levels
cellular response to nutrient levels
cellular response to phosphate starvation
xyloglucan biosynthetic process
fucose biosynthetic process
-log10FDR
12
8
4
0
SR (our data) | DN
0
2
4
6
oligosaccharide metabolic process
disaccharide metabolic process
sucrose metabolic process
-log10FDR
12
8
4
0
LF (PRJNA342750) | UP
0
10
20
30
40
response to stimulus
response to chemical
response to stress
obsolete oxidation-reduction process
response to oxygen-containing compound
pigment biosynthetic process
regulation of immune system process
water-soluble vitamin biosynthetic process
tetrapyrrole metabolic process
porphyrin-containing compound metabolic process
tetrapyrrole biosynthetic process
porphyrin-containing compound biosynthetic process
heme metabolic process
heme biosynthetic process
cobalamin biosynthetic process
siroheme metabolic process
siroheme biosynthetic process
cobalamin metabolic process
reactive nitrogen species metabolic process
nitrate assimilation
nitrate metabolic process
-log10FDR
12
8
4
0
LF (PRJNA342750) | DN
0
5
10
15
20
carbohydrate metabolic process
xenobiotic metabolic process
xenobiotic catabolic process
xenobiotic transmembrane transport
xenobiotic transport
monocarboxylic acid transport
seed coat development
mucilage metabolic process
mucilage metabolic process involved in seed coat development
mucilage biosynthetic process involved in seed coat development
-log10FDR
12
8
4
0

Supplementary Figure S2

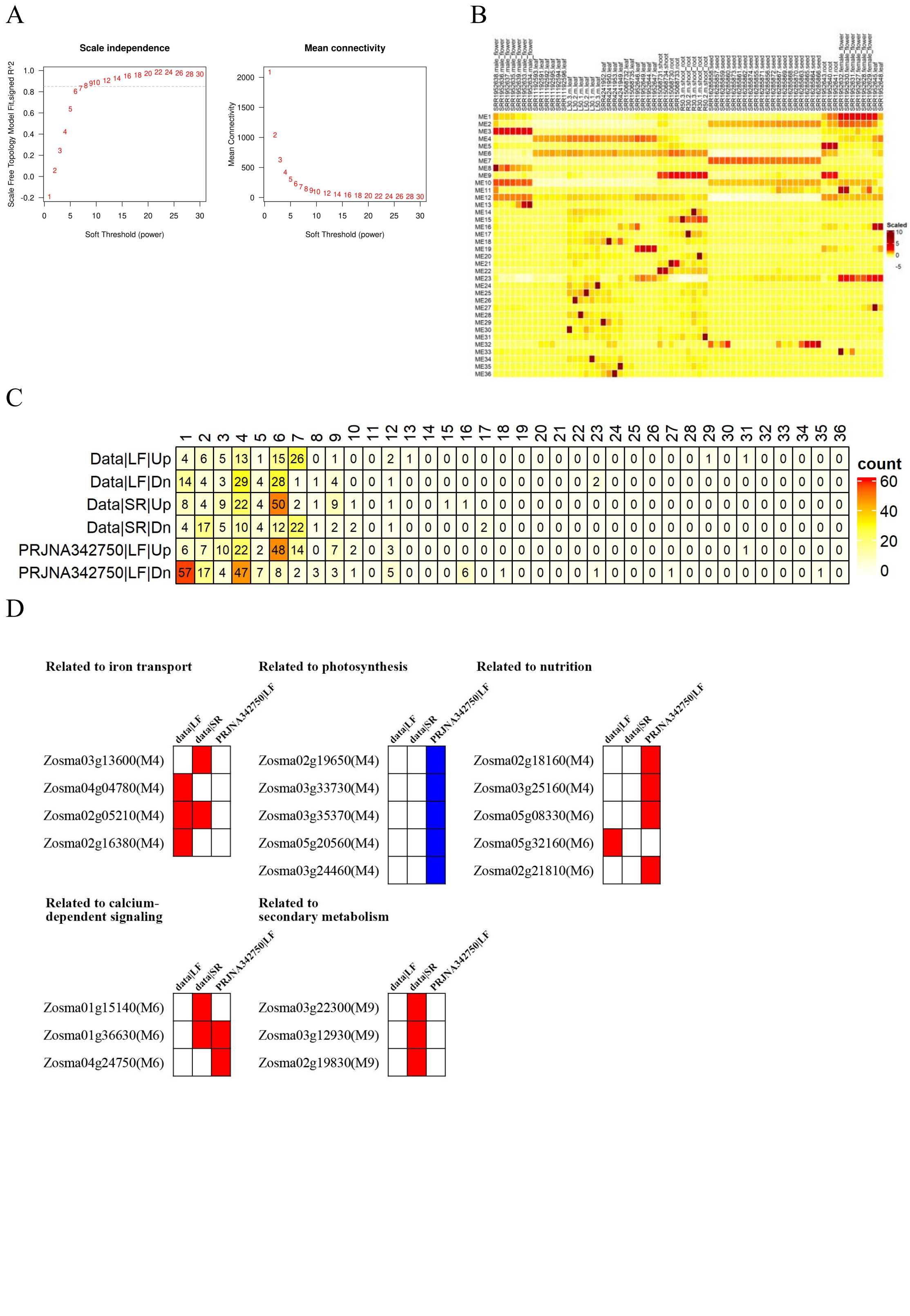

E

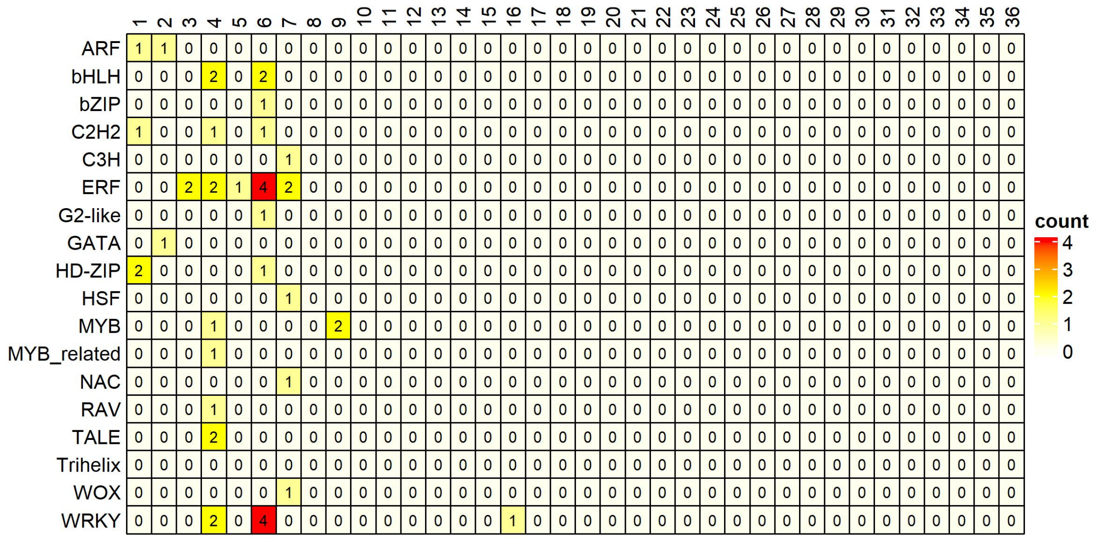
1 2 3 4 5 6 7 8 9 10 11 12 13 14 15 16 17 18 19 20 21 22 23 24 25 26 27 28 29 30 31 32 33 34 35 36
ARF 1 1 0 0 0 0 0 0 0 0 0 0 0 0 0 0 0 0 0 0 0 0 0 0 0 0 0 0 0 0 0 0 0 0 0 0
bHLH 0 0 0 2 0 2 0 0 0 0 0 0 0 0 0 0 0 0 0 0 0 0 0 0 0 0 0 0 0 0 0 0 0 0 0 0
bZIP 0 0 0 0 0 1 0 0 0 0 0 0 0 0 0 0 0 0 0 0 0 0 0 0 0 0 0 0 0 0 0 0 0 0 0 0
C2H2 1 0 0 1 0 1 0 0 0 0 0 0 0 0 0 0 0 0 0 0 0 0 0 0 0 0 0 0 0 0 0 0 0 0 0 0
C3H 0 0 0 0 0 0 1 0 0 0 0 0 0 0 0 0 0 0 0 0 0 0 0 0 0 0 0 0 0 0 0 0 0 0 0 0
ERF 0 0 2 2 1 4 2 0 0 0 0 0 0 0 0 0 0 0 0 0 0 0 0 0 0 0 0 0 0 0 0 0 0 0 0 0
G2-like 0 0 0 0 0 1 0 0 0 0 0 0 0 0 0 0 0 0 0 0 0 0 0 0 0 0 0 0 0 0 0 0 0 0 0 0
GATA 0 1 0 0 0 0 0 0 0 0 0 0 0 0 0 0 0 0 0 0 0 0 0 0 0 0 0 0 0 0 0 0 0 0 0 0
HD-ZIP 2 0 0 0 0 1 0 0 0 0 0 0 0 0 0 0 0 0 0 0 0 0 0 0 0 0 0 0 0 0 0 0 0 0 0 0
HSF 0 0 0 0 0 0 1 0 0 0 0 0 0 0 0 0 0 0 0 0 0 0 0 0 0 0 0 0 0 0 0 0 0 0 0 0
MYB 0 0 0 1 0 0 0 0 2 0 0 0 0 0 0 0 0 0 0 0 0 0 0 0 0 0 0 0 0 0 0 0 0 0 0 0
MYB_related 0 0 0 1 0 0 0 0 0 0 0 0 0 0 0 0 0 0 0 0 0 0 0 0 0 0 0 0 0 0 0 0 0 0 0 0
NAC 0 0 0 0 0 0 1 0 0 0 0 0 0 0 0 0 0 0 0 0 0 0 0 0 0 0 0 0 0 0 0 0 0 0 0 0
RAV 0 0 0 1 0 0 0 0 0 0 0 0 0 0 0 0 0 0 0 0 0 0 0 0 0 0 0 0 0 0 0 0 0 0 0 0
TALE 0 0 0 2 0 0 0 0 0 0 0 0 0 0 0 0 0 0 0 0 0 0 0 0 0 0 0 0 0 0 0 0 0 0 0 0
Trihelix 0 0 0 0 0 0 0 0 0 0 0 0 0 0 0 0 0 0 0 0 0 0 0 0 0 0 0 0 0 0 0 0 0 0 0 0
WOX 0 0 0 0 0 0 1 0 0 0 0 0 0 0 0 0 0 0 0 0 0 0 0 0 0 0 0 0 0 0 0 0 0 0 0 0
WRKY 0 0 0 2 0 4 0 0 0 0 0 0 0 0 0 1 0 0 0 0 0 0 0 0 0 0 0 0 0 0 0 0 0 0 0 0
count
4
3
2
1
0

F

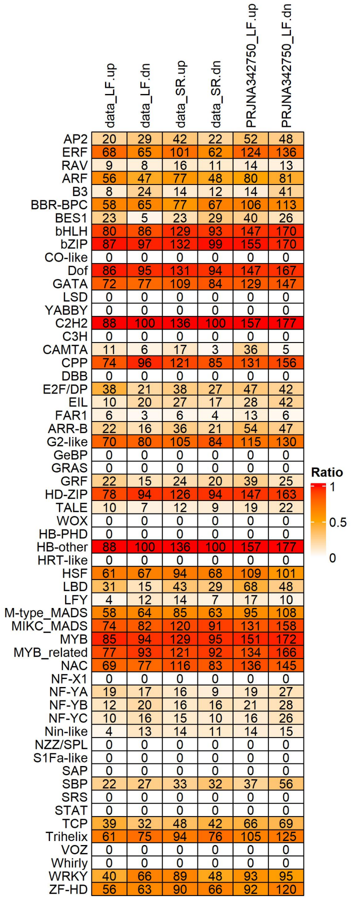
data_LF.up data_LF.dn data_SR.up data_SR.dn PRJNA342750_LF.up PRJNA342750_LF.dn
AP2 20 29 42 22 52 48
ERF 68 65 101 62 124 136
RAV 9 8 16 11 14 13
ARF 56 47 77 48 80 81
B3 8 24 14 12 14 41
BBR-BPC 58 65 77 67 106 113
BES1 23 5 23 29 40 26
bHLH 80 86 129 93 147 170
bZIP 87 97 132 99 155 170
CO-like 0 0 0 0 0 0
Dof 86 95 131 94 147 167
GATA 72 77 109 84 129 147
LSD 0 0 0 0 0 0
YABBY 0 0 0 0 0 0
C2H2 88 100 136 100 157 177
C3H 0 0 0 0 0 0
CAMTA 11 6 17 3 36 5
CPP 74 96 121 85 131 156
DBB 0 0 0 0 0 0
E2F/DP 38 21 38 27 47 42
EIL 10 20 27 17 28 42
FAR1 6 3 6 4 13 6
ARR-B 22 16 36 21 54 47
G2-like 70 80 105 84 115 130
GeBP 0 0 0 0 0 0
GRAS 0 0 0 0 0 0
GRF 22 15 24 20 39 25
HD-ZIP 78 94 126 94 147 163
TALE 10 7 12 9 19 22
WOX 0 0 0 0 0 0
HB-PHD 0 0 0 0 0 0
HB-other 88 100 136 100 157 177
HRT-like 0 0 0 0 0 0
HSF 61 67 94 68 109 101
LBD 31 15 43 29 68 48
LFY 4 12 14 7 17 10
M-type_MADS 58 64 85 63 95 108
MIKC_MADS 74 82 120 91 131 158
MYB 85 94 129 95 151 172
MYB_related 77 93 121 92 134 166
NAC 69 77 116 83 136 145
NF-X1 0 0 0 0 0 0
NF-YA 19 17 16 9 19 27
NF-YB 12 20 16 16 21 28
NF-YC 10 16 15 10 16 26
Nin-like 4 13 14 11 14 15
NZZ/SPL 0 0 0 0 0 0
S1Fa-like 0 0 0 0 0 0
SAP 0 0 0 0 0 0
SBP 22 27 33 32 37 56
SRS 0 0 0 0 0 0
STAT 0 0 0 0 0 0
TCP 39 32 48 42 66 69
Trihelix 61 75 94 76 105 125
VOZ 0 0 0 0 0 0
Whirly 0 0 0 0 0 0
WRKY 40 66 89 48 93 95
ZF-HD 56 63 90 66 92 120
Ratio
1
0.5
0

G

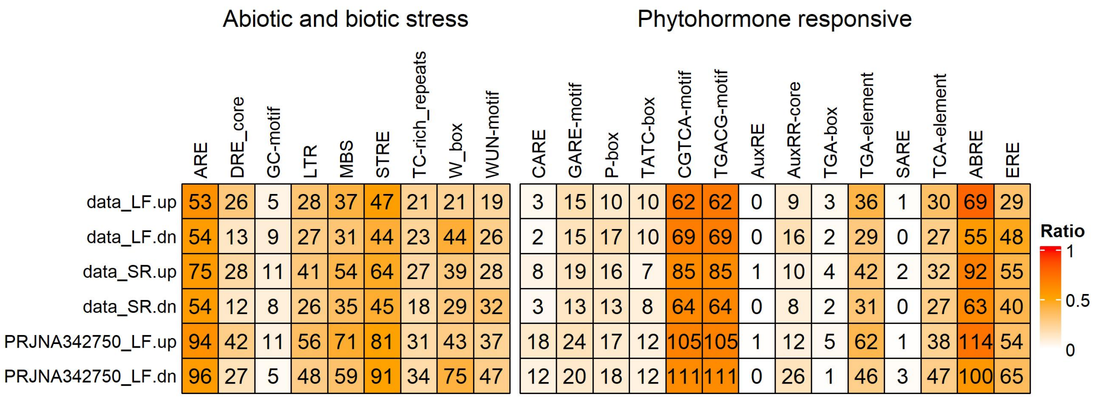
Abiotic and biotic stress
Phytohormone responsive
ARE
DRE_core
GC-motif
LTR
MBS
STRE
TC-rich_repeats
W_box
WUN-motif
CARE
GARE-motif
P-box
TATC-box
CGTCA-motif
TGACG-motif
AuxRE
AuxRR-core
TGA-box
TGA-element
SARE
TCA-element
ABRE
ERE
data_LF.up 53 26 5 28 37 47 21 21 19 3 15 10 10 62 62 0 9 3 36 1 30 69 29
data_LF.dn 54 13 9 27 31 44 23 44 26 2 15 17 10 69 69 0 16 2 29 0 27 55 48
data_SR.up 75 28 11 41 54 64 27 39 28 8 19 16 7 85 85 1 10 4 42 2 32 92 55
data_SR.dn 54 12 8 26 35 45 18 29 32 3 13 13 8 64 64 0 8 2 31 0 27 63 40
PRJNA342750_LF.up 94 42 11 56 71 81 31 43 37 18 24 17 12 105 105 1 12 5 62 1 38 114 54
PRJNA342750_LF.dn 96 27 5 48 59 91 34 75 47 12 20 18 12 111 111 0 26 1 46 3 47 100 65
Ratio
1
0.5
0

Supplementary Figure S3

A

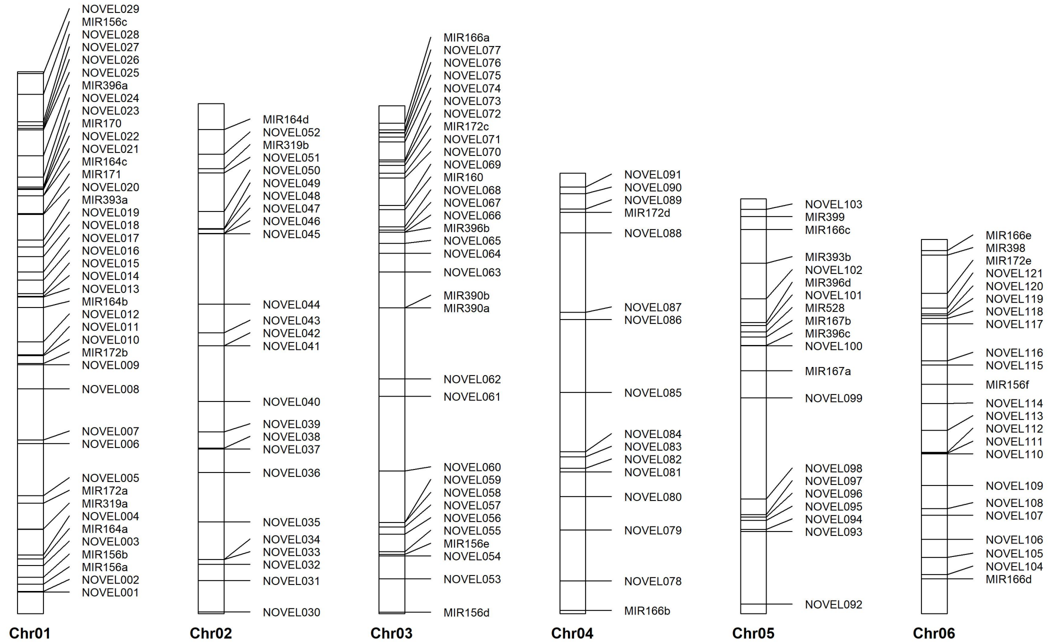

B

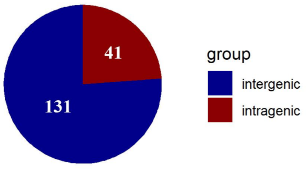

C

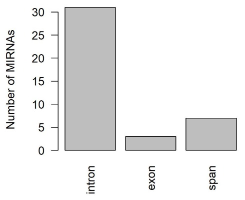

D

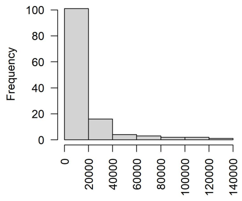

E

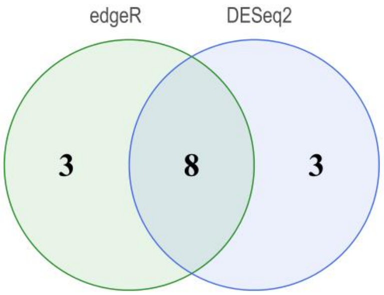

F

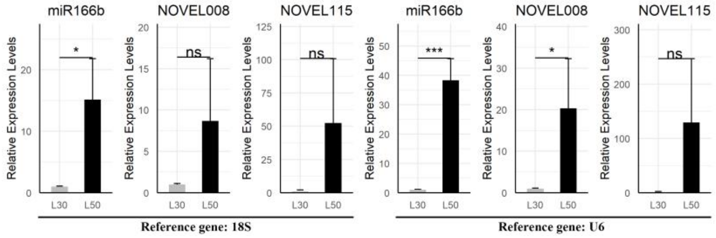

Supplementary Figure S4

A B

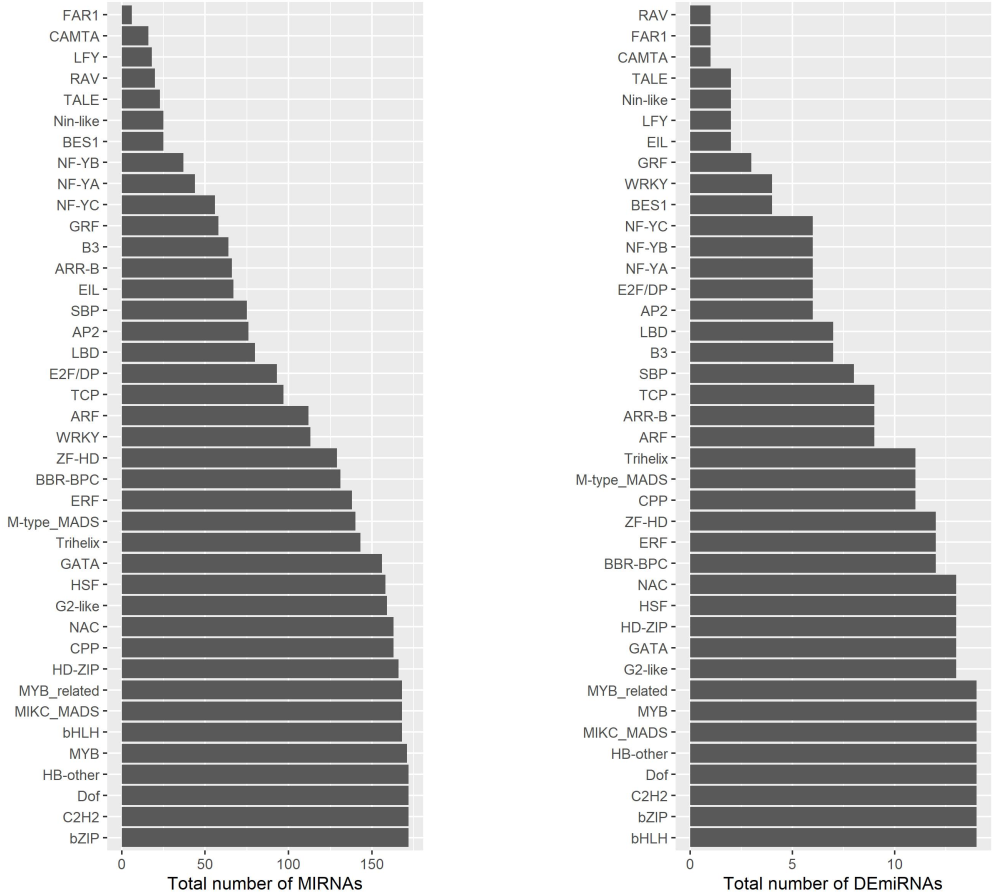

C

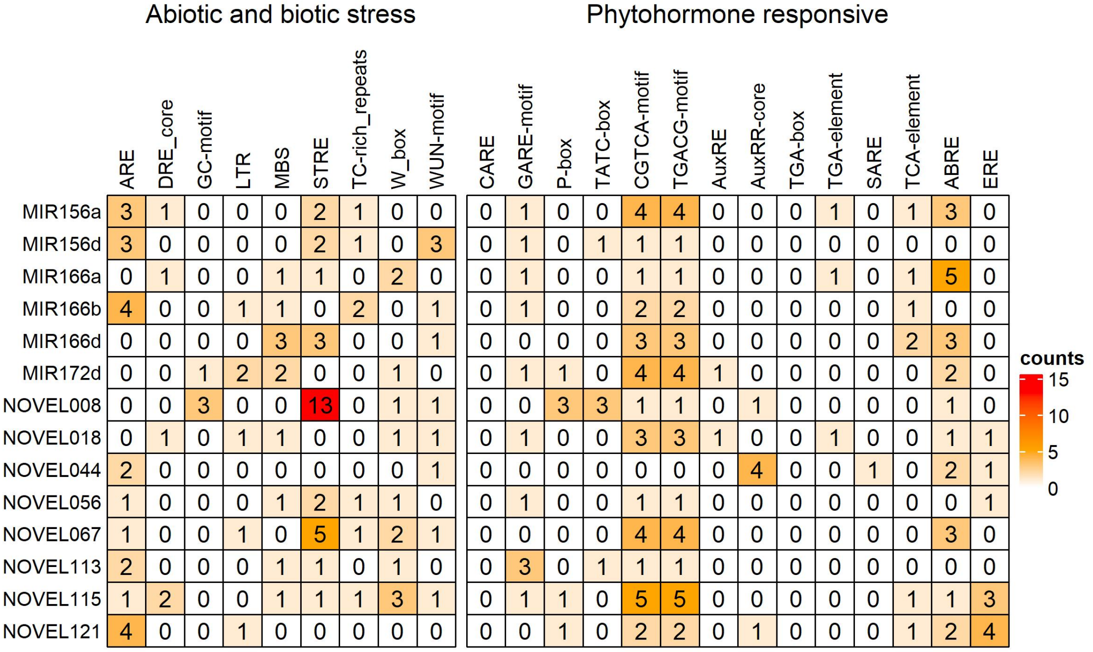

Supplementary Figure S5

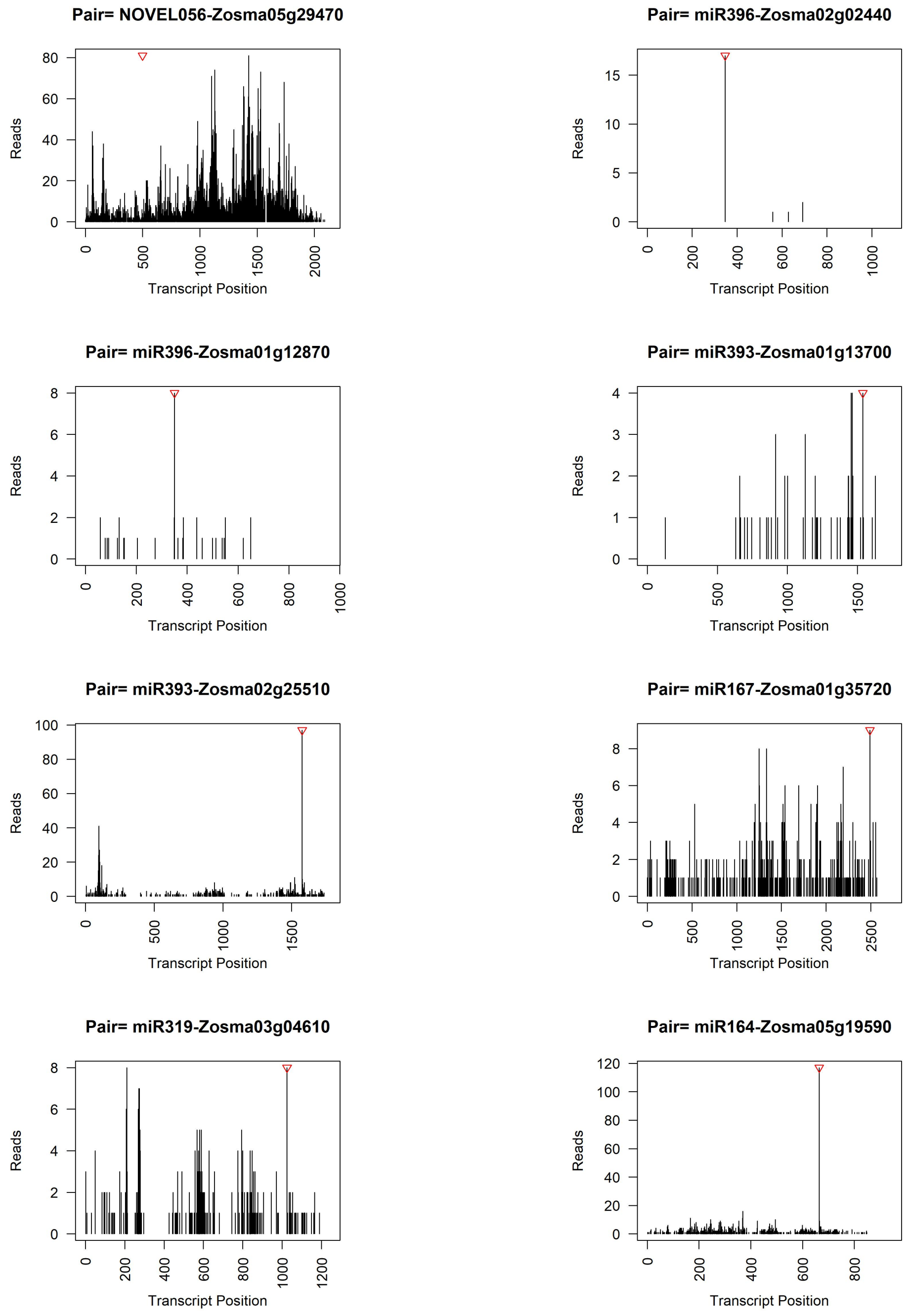

Supplementary Figure S6

A

Zosma01g16060

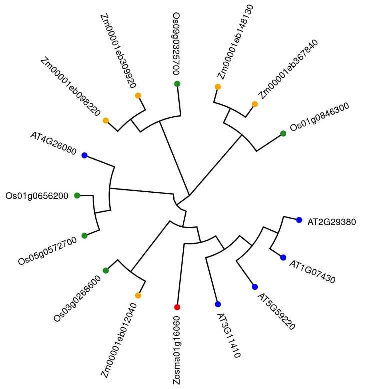

Zosma05g33490

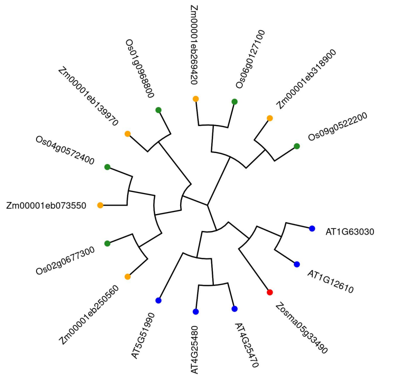

Zosma03g37150

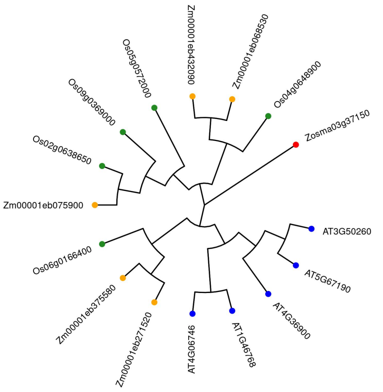

Zosma05g03710

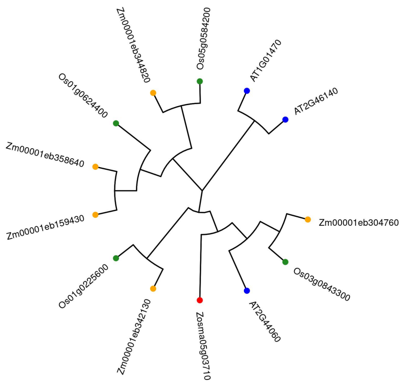

Zosma03g14850

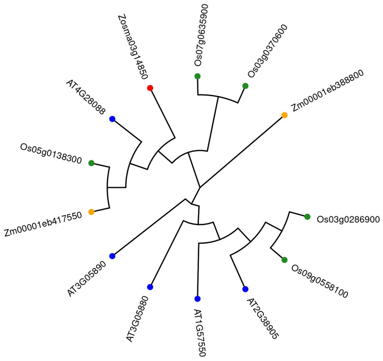

B

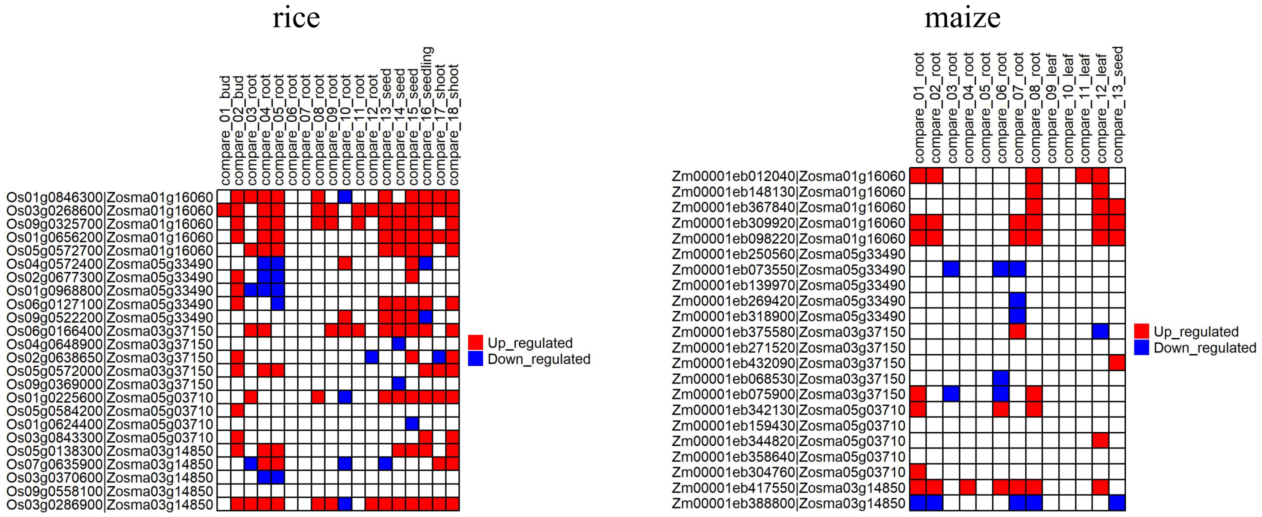
rice
maize
compare_01_bud
compare_02_bud
compare_03_root
compare_04_root
compare_05_root
compare_06_root
compare_07_root
compare_08_root
compare_09_root
compare_10_root
compare_11_root
compare_12_root
compare_13_seed
compare_14_seed
compare_15_seed
compare_16_seedling
compare_17_shoot
compare_18_shoot
Os01g0846300|Zosma01g16060
Os03g0268600|Zosma01g16060
Os09g0325700|Zosma01g16060
Os01g0656200|Zosma01g16060
Os05g0572700|Zosma01g16060
Os04g0572400|Zosma05g33490
Os02g0677300|Zosma05g33490
Os01g0968800|Zosma05g33490
Os06g0127100|Zosma05g33490
Os09g0522200|Zosma05g33490
Os06g0166400|Zosma03g37150
Os04g0648900|Zosma03g37150
Os02g0638650|Zosma03g37150
Os05g0572000|Zosma03g37150
Os09g0369000|Zosma03g37150
Os01g0225600|Zosma05g03710
Os05g0584200|Zosma05g03710
Os01g0624400|Zosma05g03710
Os03g0843300|Zosma05g03710
Os05g0138300|Zosma03g14850
Os07g0635900|Zosma03g14850
Os03g0370600|Zosma03g14850
Os09g0558100|Zosma03g14850
Os03g0286900|Zosma03g14850
Up_regulated
Down_regulated
compare_01_root
compare_02_root
compare_03_root
compare_04_root
compare_05_root
compare_06_root
compare_07_root
compare_08_root
compare_09_leaf
compare_10_leaf
compare_11_leaf
compare_12_leaf
compare_13_seed
Zm00001eb012040|Zosma01g16060
Zm00001eb148130|Zosma01g16060
Zm00001eb367840|Zosma01g16060
Zm00001eb309920|Zosma01g16060
Zm00001eb098220|Zosma01g16060
Zm00001eb250560|Zosma05g33490
Zm00001eb073550|Zosma05g33490
Zm00001eb139970|Zosma05g33490
Zm00001eb269420|Zosma05g33490
Zm00001eb318900|Zosma05g33490
Zm00001eb375580|Zosma03g37150
Zm00001eb271520|Zosma03g37150
Zm00001eb432090|Zosma03g37150
Zm00001eb068530|Zosma03g37150
Zm00001eb075900|Zosma03g37150
Zm00001eb342130|Zosma05g03710
Zm00001eb159430|Zosma05g03710
Zm00001eb344820|Zosma05g03710
Zm00001eb358640|Zosma05g03710
Zm00001eb304760|Zosma05g03710
Zm00001eb417550|Zosma03g14850
Zm00001eb388800|Zosma03g14850
Up_regulated
Down_regulated

C

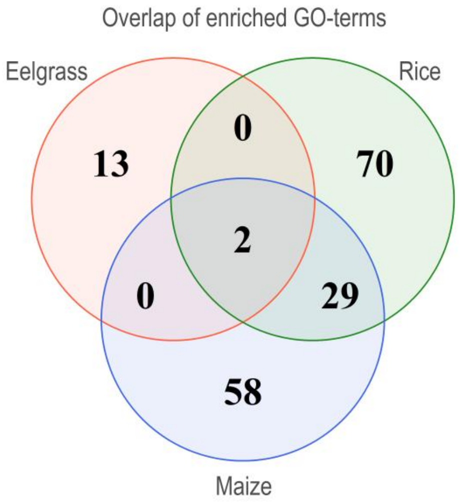
Overlap of enriched GO-terms
Eelgrass
Rice
Maize
13
0
70
2
0
29
58

Supplementary Figure S7

A

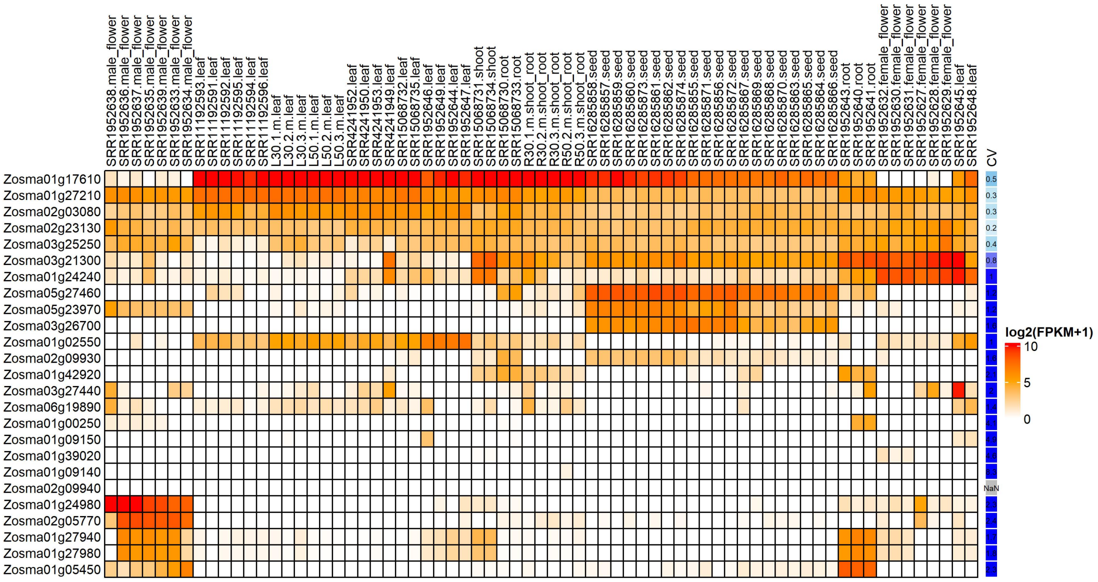

B

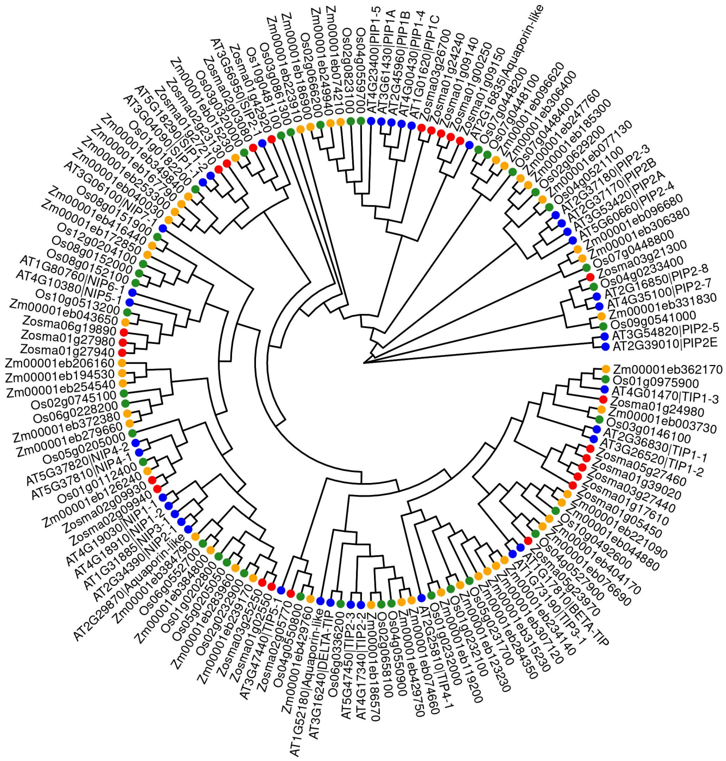

C

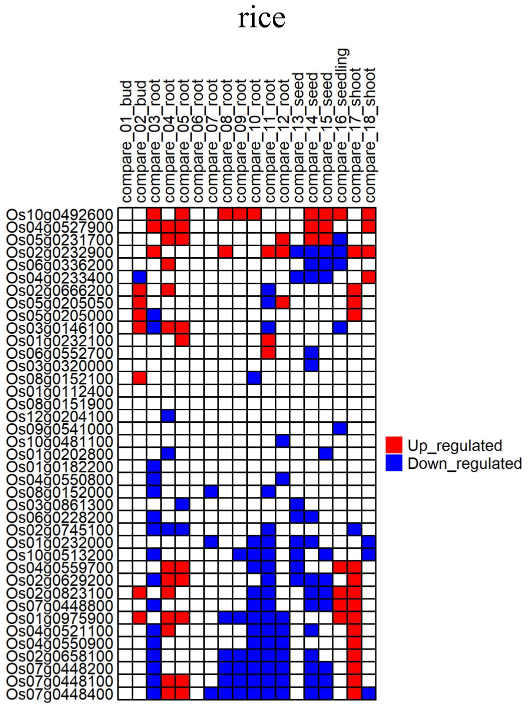
rice
compare_01_bud
compare_02_bud
compare_03_root
compare_04_root
compare_05_root
compare_06_root
compare_07_root
compare_08_root
compare_09_root
compare_10_root
compare_11_root
compare_12_root
compare_13_seed
compare_14_seed
compare_15_seed
compare_16_seedling
compare_17_shoot
compare_18_shoot
Os10g0492600
Os04g0527900
Os05g0231700
Os02g0232900
Os06g0336200
Os04g0233400
Os02g0666200
Os05g0205050
Os05g0205000
Os03g0146100
Os01g0232100
Os06g0552700
Os03g0320000
Os08g0152100
Os01g0112400
Os08g0151900
Os12g0204100
Os09g0541000
Os10g0481100
Os01g0202800
Os01g0182200
Os04g0550800
Os08g0152000
Os03g0861300
Os06g0228200
Os02g0745100
Os01g0232000
Os10g0513200
Os04g0559700
Os02g0629200
Os02g0823100
Os07g0448800
Os01g0975900
Os04g0521100
Os04g0550900
Os02g0658100
Os07g0448200
Os07g0448100
Os07g0448400
Up_regulated
Down_regulated

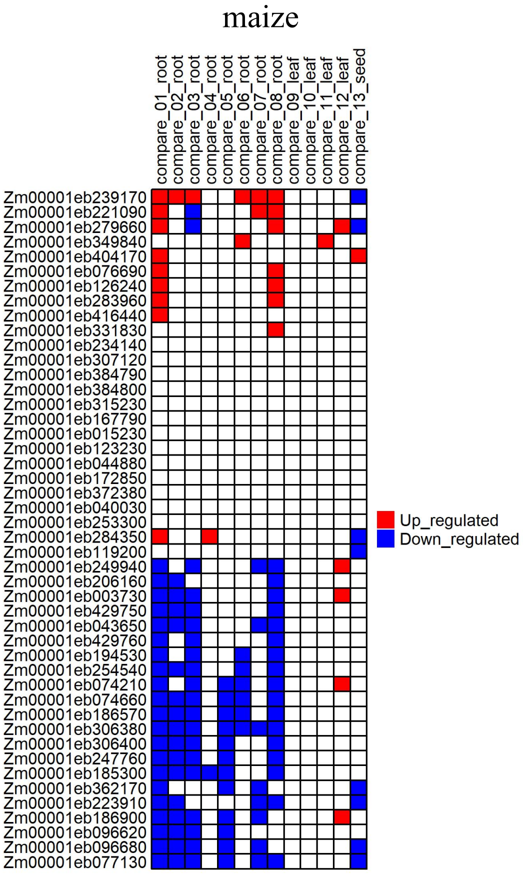
maize
compare_01_root
compare_02_root
compare_03_root
compare_04_root
compare_05_root
compare_06_root
compare_07_root
compare_08_root
compare_09_leaf
compare_10_leaf
compare_11_leaf
compare_12_leaf
compare_13_seed
Zm00001eb239170
Zm00001eb221090
Zm00001eb279660
Zm00001eb349840
Zm00001eb404170
Zm00001eb076690
Zm00001eb126240
Zm00001eb283960
Zm00001eb416440
Zm00001eb331830
Zm00001eb234140
Zm00001eb307120
Zm00001eb384790
Zm00001eb384800
Zm00001eb315230
Zm00001eb167790
Zm00001eb015230
Zm00001eb123230
Zm00001eb044880
Zm00001eb172850
Zm00001eb372380
Zm00001eb040030
Zm00001eb253300
Zm00001eb284350
Zm00001eb119200
Zm00001eb249940
Zm00001eb206160
Zm00001eb003730
Zm00001eb429750
Zm00001eb043650
Zm00001eb429760
Zm00001eb194530
Zm00001eb254540
Zm00001eb074210
Zm00001eb074660
Zm00001eb186570
Zm00001eb306380
Zm00001eb306400
Zm00001eb247760
Zm00001eb185300
Zm00001eb362170
Zm00001eb223910
Zm00001eb186900
Zm00001eb096620
Zm00001eb096680
Zm00001eb077130
Up_regulated
Down_regulated

Supplementary Figure S8

A

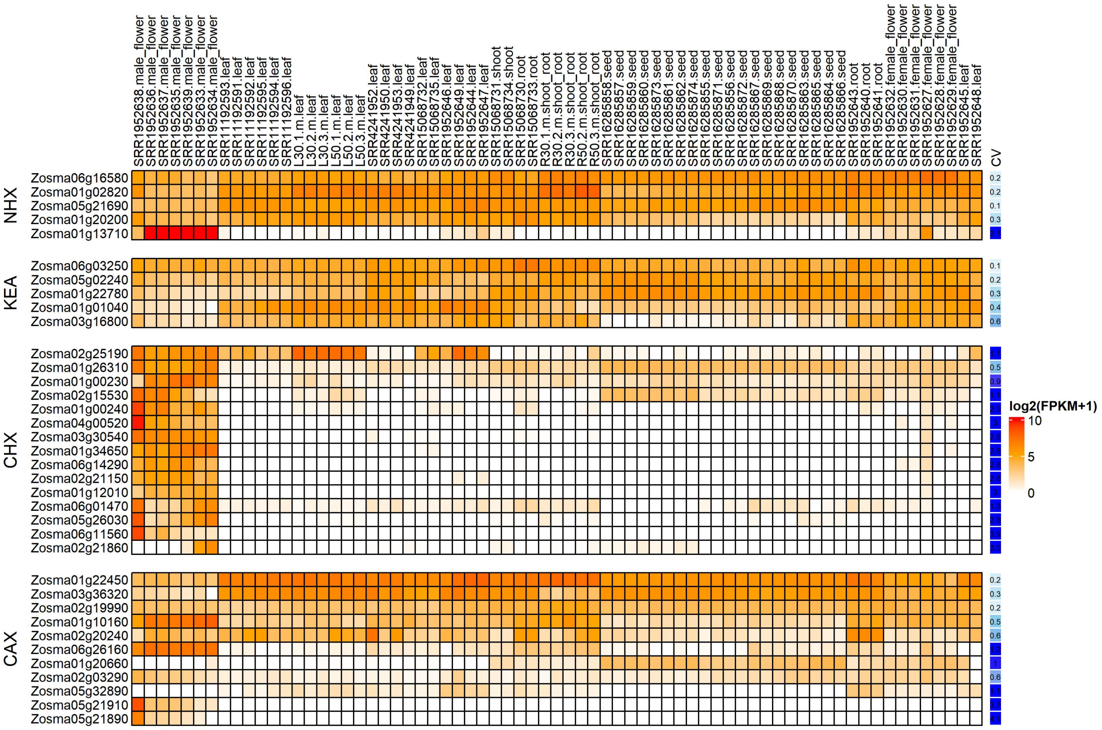

B

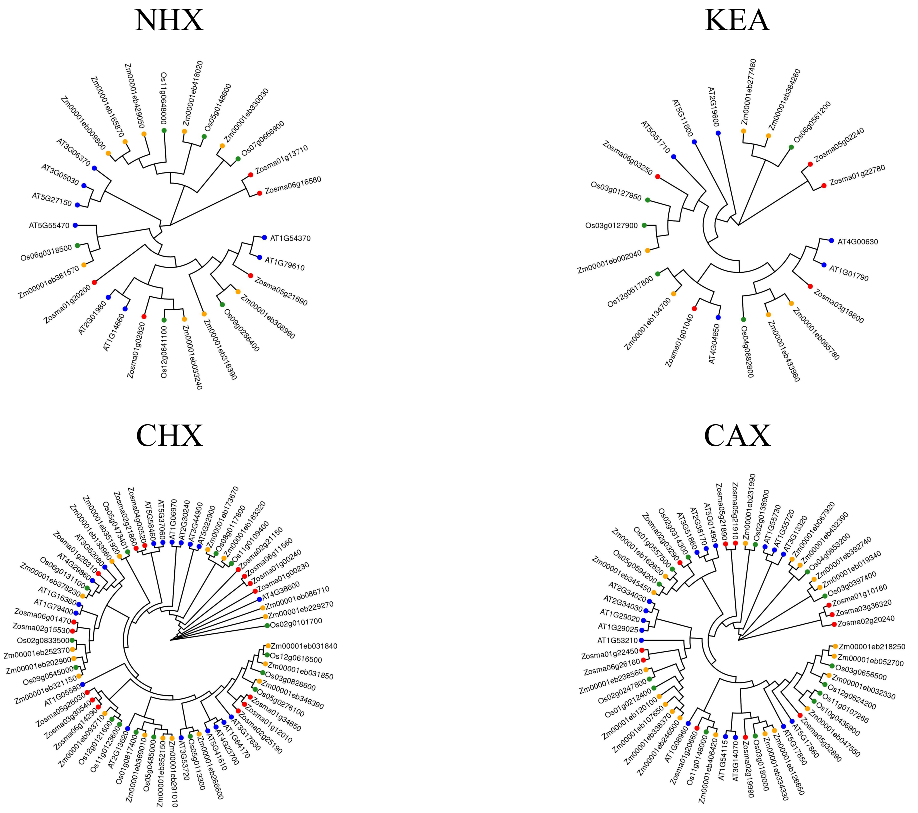

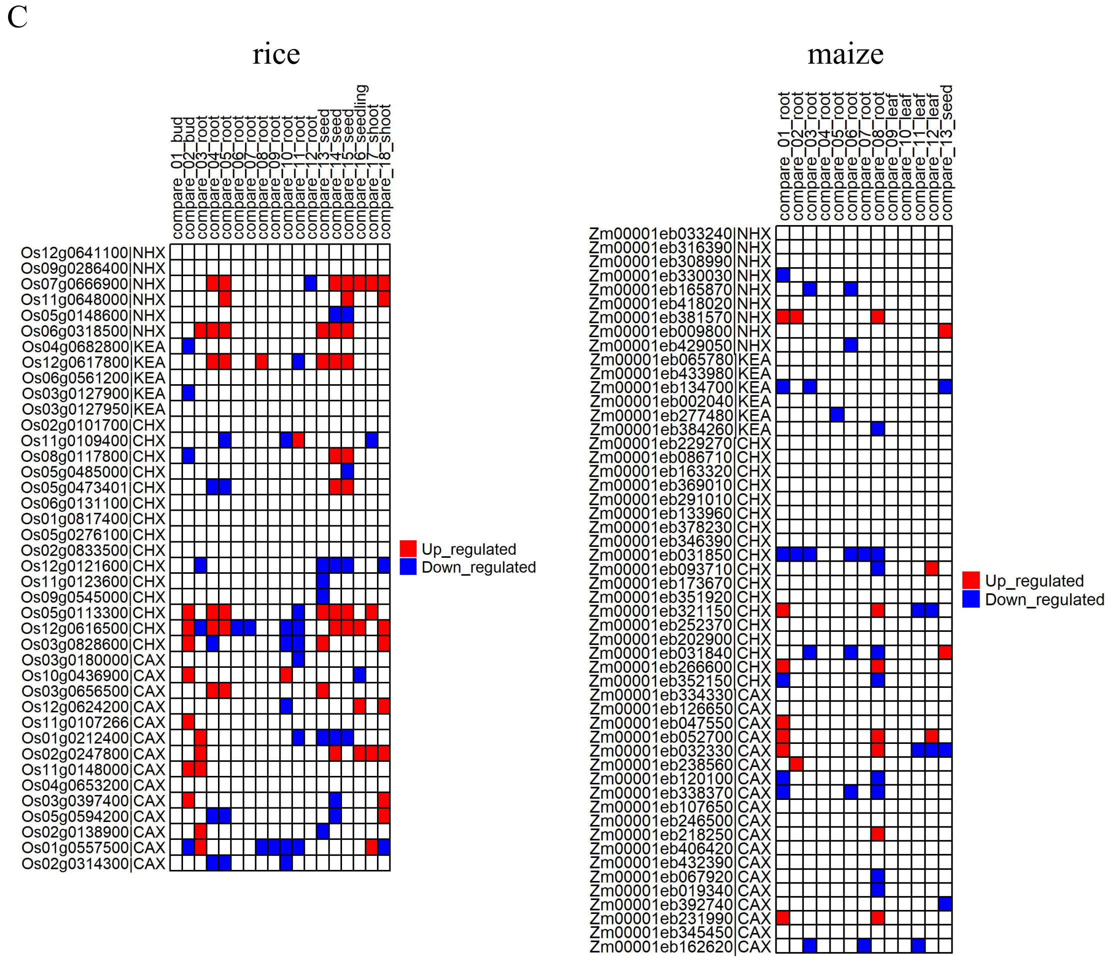

C
rice
maize
Up_regulated
Down_regulated

Supplementary Figure S9

A

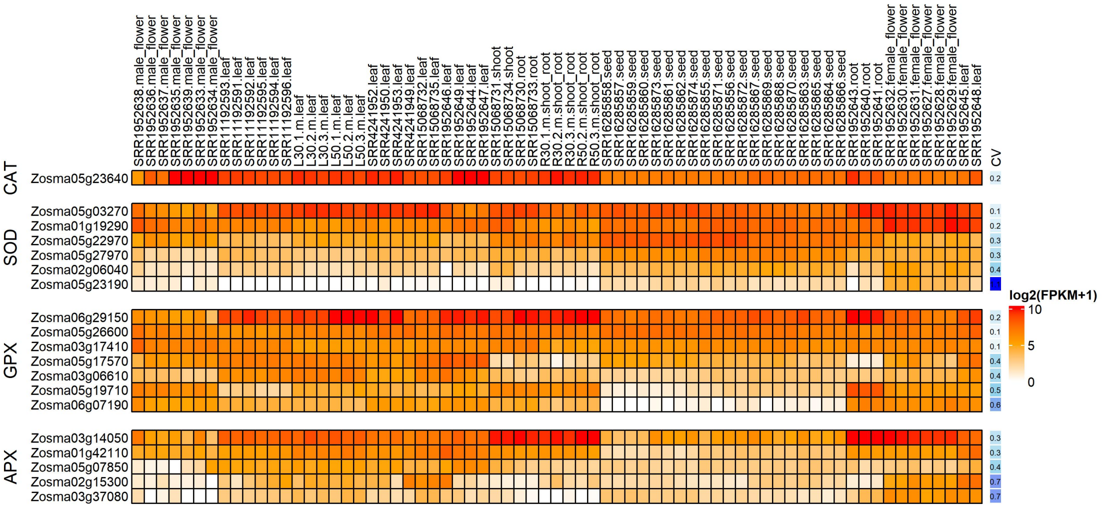

B

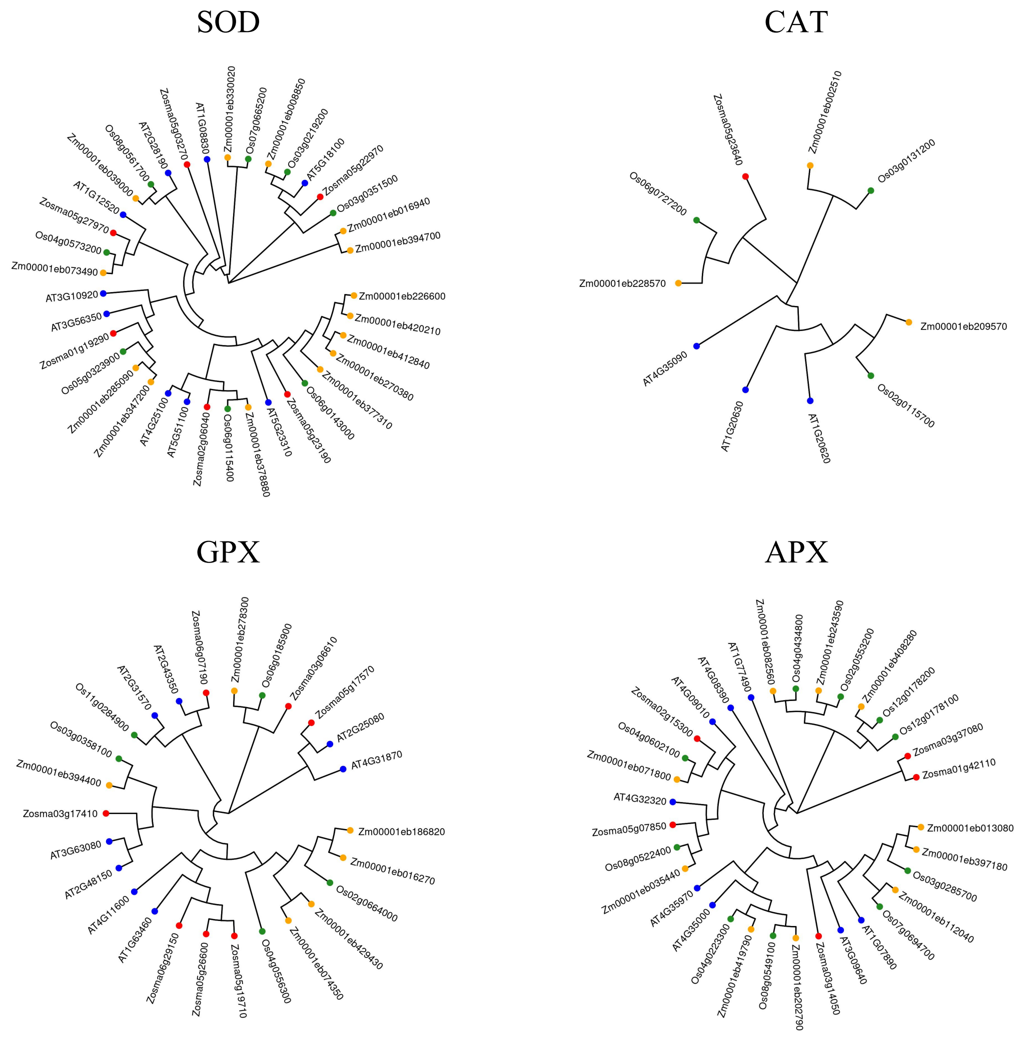

C

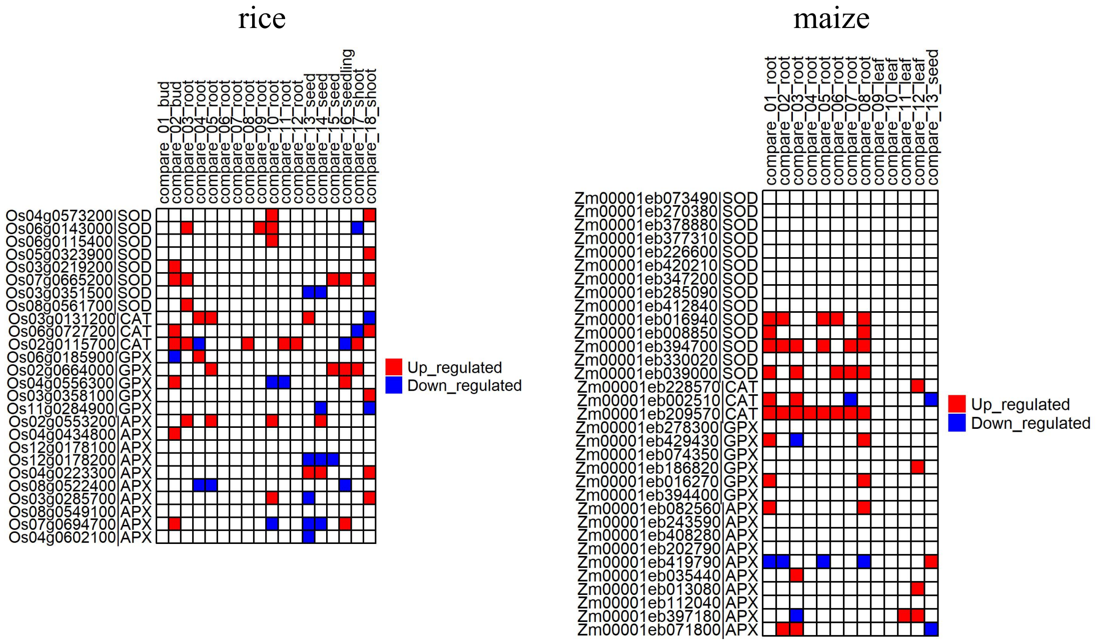

Supplementary Figure S10

A

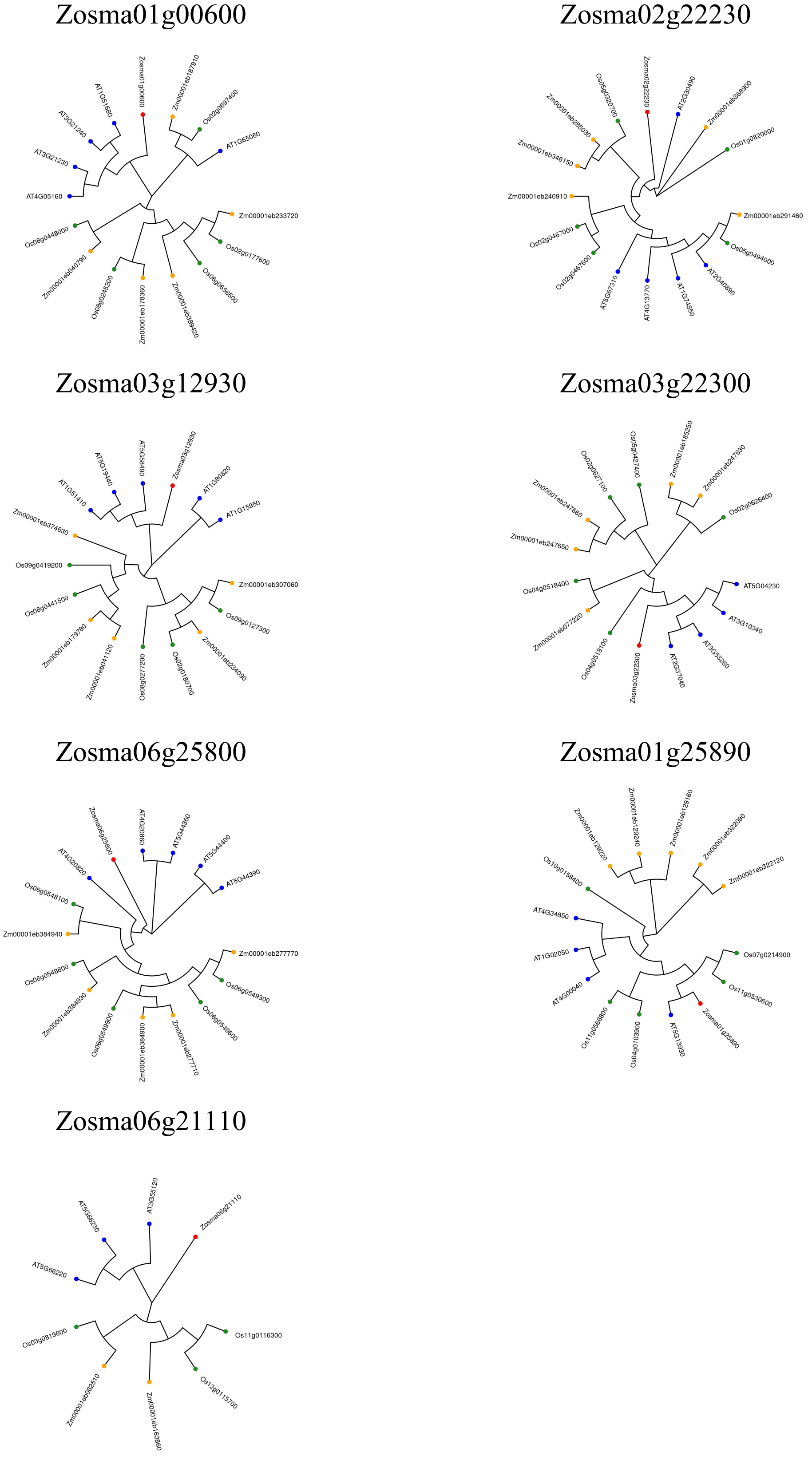

B

rice

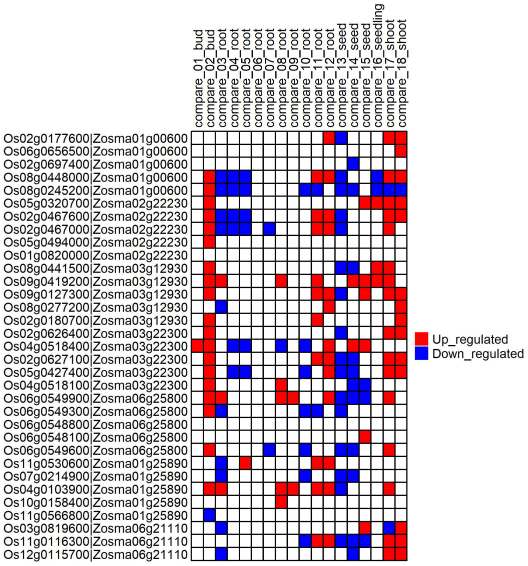
compare_01_bud
compare_02_bud
compare_03_root
compare_04_root
compare_05_root
compare_06_root
compare_07_root
compare_08_root
compare_09_root
compare_10_root
compare_11_root
compare_12_root
compare_13_seed
compare_14_seed
compare_15_seed
compare_16_seedling
compare_17_shoot
compare_18_shoot
Os02g0177600|Zosma01g00600
Os06g0656500|Zosma01g00600
Os02g0697400|Zosma01g00600
Os08g0448000|Zosma01g00600
Os08g0245200|Zosma01g00600
Os05g0320700|Zosma02g22230
Os02g0467600|Zosma02g22230
Os02g0467000|Zosma02g22230
Os05g0494000|Zosma02g22230
Os01g0820000|Zosma02g22230
Os08g0441500|Zosma03g12930
Os09g0419200|Zosma03g12930
Os09g0127300|Zosma03g12930
Os08g0277200|Zosma03g12930
Os02g0180700|Zosma03g12930
Os02g0626400|Zosma03g22300
Os04g0518400|Zosma03g22300
Os02g0627100|Zosma03g22300
Os05g0427400|Zosma03g22300
Os04g0518100|Zosma03g22300
Os06g0549900|Zosma06g25800
Os06g0549300|Zosma06g25800
Os06g0548800|Zosma06g25800
Os06g0548100|Zosma06g25800
Os06g0549600|Zosma06g25800
Os11g0530600|Zosma01g25890
Os07g0214900|Zosma01g25890
Os04g0103900|Zosma01g25890
Os10g0158400|Zosma01g25890
Os11g0566800|Zosma01g25890
Os03g0819600|Zosma06g21110
Os11g0116300|Zosma06g21110
Os12g0115700|Zosma06g21110
Up_regulated
Down_regulated

maize

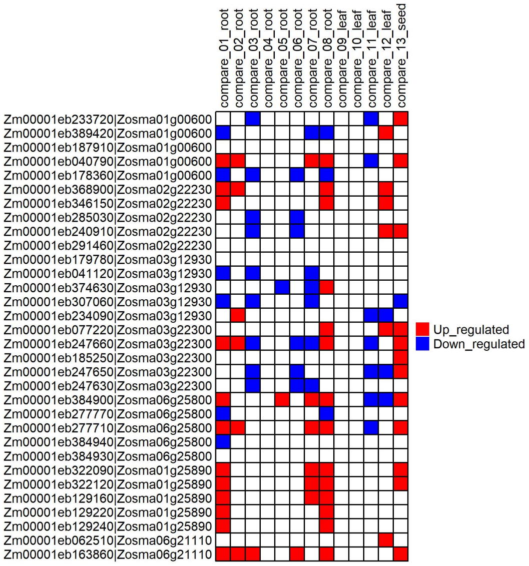
compare_01_root
compare_02_root
compare_03_root
compare_04_root
compare_05_root
compare_06_root
compare_07_root
compare_08_root
compare_09_leaf
compare_10_leaf
compare_11_leaf
compare_12_leaf
compare_13_seed
Zm00001eb233720|Zosma01g00600
Zm00001eb389420|Zosma01g00600
Zm00001eb187910|Zosma01g00600
Zm00001eb040790|Zosma01g00600
Zm00001eb178360|Zosma01g00600
Zm00001eb368900|Zosma02g22230
Zm00001eb346150|Zosma02g22230
Zm00001eb285030|Zosma02g22230
Zm00001eb240910|Zosma02g22230
Zm00001eb291460|Zosma02g22230
Zm00001eb179780|Zosma03g12930
Zm00001eb041120|Zosma03g12930
Zm00001eb374630|Zosma03g12930
Zm00001eb307060|Zosma03g12930
Zm00001eb234090|Zosma03g12930
Zm00001eb077220|Zosma03g22300
Zm00001eb247660|Zosma03g22300
Zm00001eb185250|Zosma03g22300
Zm00001eb247650|Zosma03g22300
Zm00001eb247630|Zosma03g22300
Zm00001eb384900|Zosma06g25800
Zm00001eb277770|Zosma06g25800
Zm00001eb277710|Zosma06g25800
Zm00001eb384940|Zosma06g25800
Zm00001eb384930|Zosma06g25800
Zm00001eb322090|Zosma01g25890
Zm00001eb322120|Zosma01g25890
Zm00001eb129160|Zosma01g25890
Zm00001eb129220|Zosma01g25890
Zm00001eb129240|Zosma01g25890
Zm00001eb062510|Zosma06g21110
Zm00001eb163860|Zosma06g21110
Up_regulated
Down_regulated

Supplementary Figure S11

A

Zosma01g20220|ABA

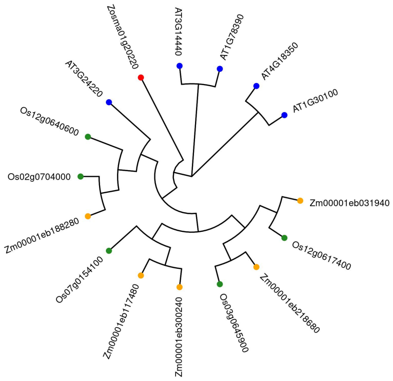

Zosma03g27730|ABA

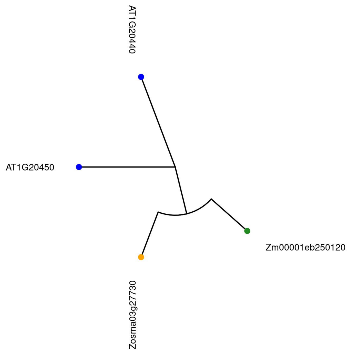

Zosma03g25920|ABA

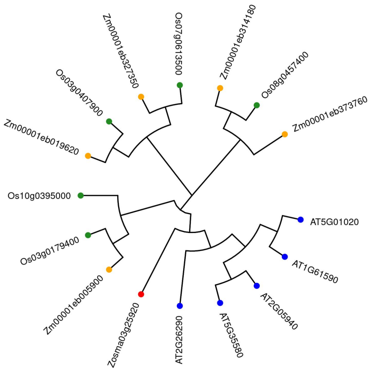

Zosma02g21670,Zosma01g41250|PP2C|ABA

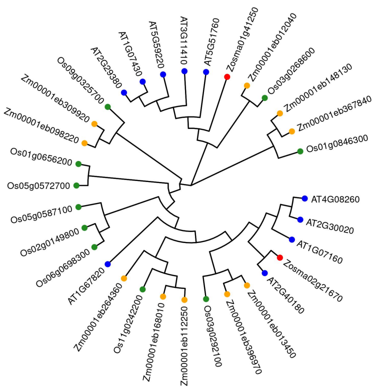

Zosma01g30620|JA

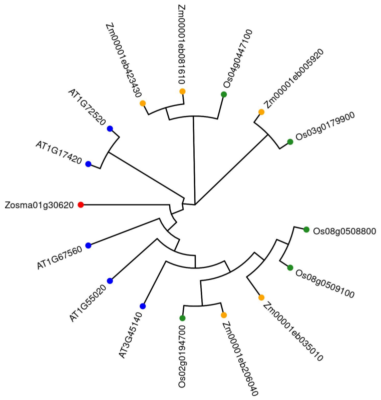

Zosma05g15520|JA

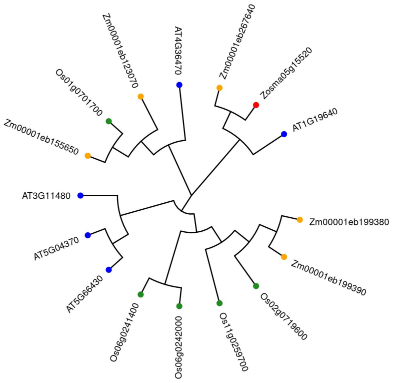

Zosma06g28020|JA

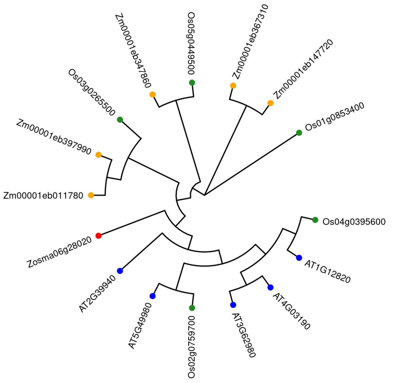

Zosma03g17780|Auxin

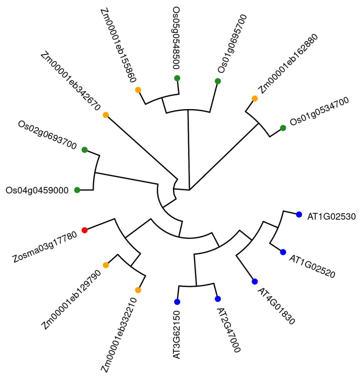

Zosma05g12790|Auxin

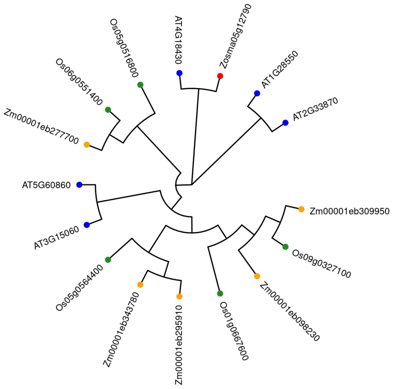

Zosma03g35130|BR

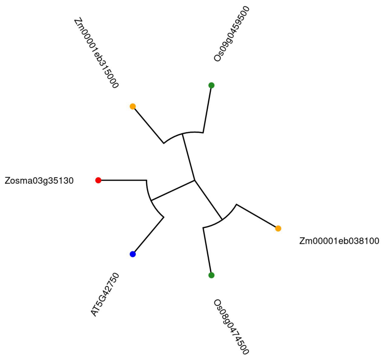

Zosma03g30090|BR

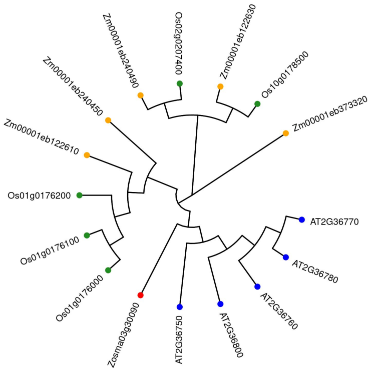

Zosma06g28530,Zosma01g36380|CK

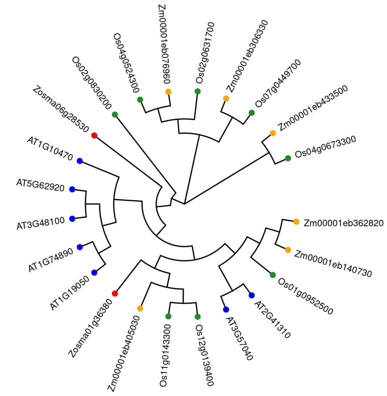

B

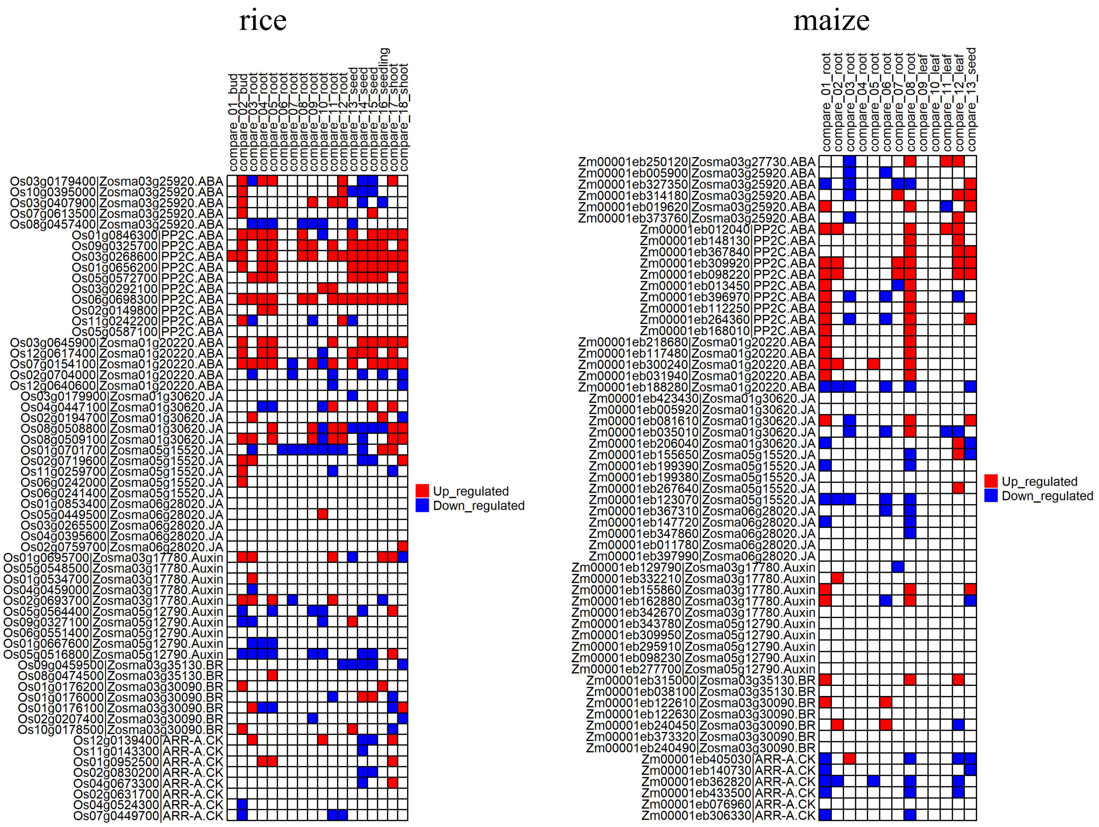

C

Homologous gene list

Phytohormone	Genes in eelgrass	Homologous gene	Reference
ABA	Zosma03g27730	RAB18（AT5G66400）	10.1016/j.bbrc.2019.01.095
ABA	Zosma03g25920	ARSK1（AT2G26290）	10.1046/j.1365-313x.1995.08010037.x
ABA	Zosma01g41250	HAI1（AT5G59220）<br>HAI2（AT1G07430）	10.1007/s11033-009-9601-8<br>10.1104/pp.112.202408
ABA	Zosma02g21670	PP2C5（AT2G40180）<br>AP2C1（AT2G30020）	10.1104/pp.110.156109<br>10.1105/tpc.106.049585
JA	Zosma01g30620	LOX3（AT1G17420）	10.1016/j.jplph.2014.07.006
JA	Zosma05g15520	JMT（AT1G19640）	10.1073/pnas.081557298
JA	Zosma06g28020	COI1（AT2G39940）	10.1111/tpj.15464
Auxin	Zosma03g17780	PGP4（AT2G47000）	10.1105/tpc.107.054288
Auxin	Zosma05g12790	ARA-2（AT1G06400）	10.1007/s11103-009-9460-7
BR	Zosma03g35130	BKI1（AT5G42750）	10.1111/pce.13064
BR	Zosma03g30090	DOGT1（AT2G36800）	10.1073/pnas.0504279102
CK	Zosma06g28530<br>Zosma01g36380	ARR-A（KO: K14492）	10.1111/tpj.12098<br>10.1271/bbb.80402

Supplementary Figure S12

A

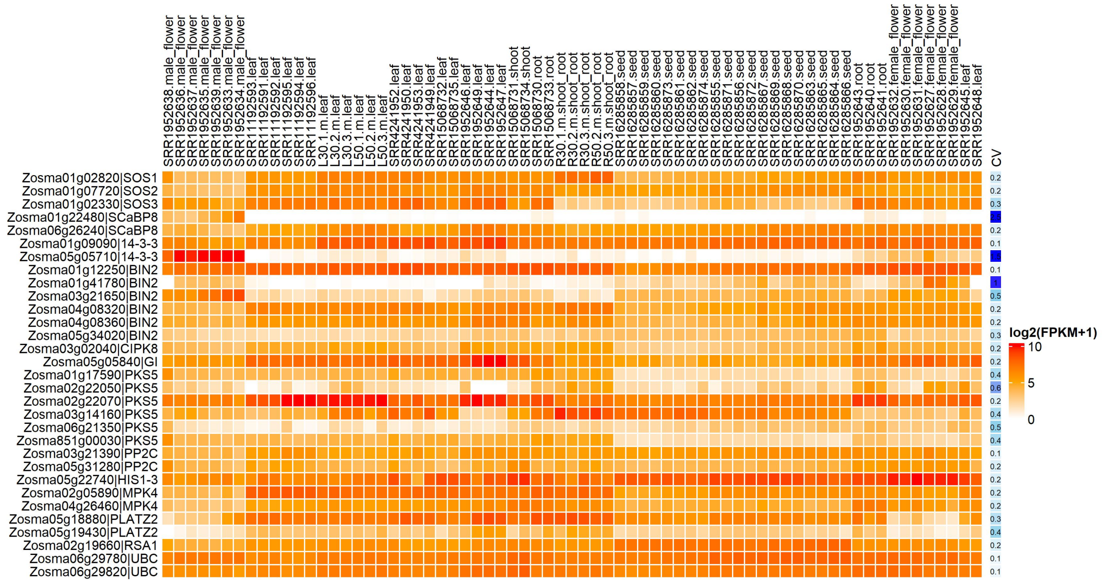

B

rice

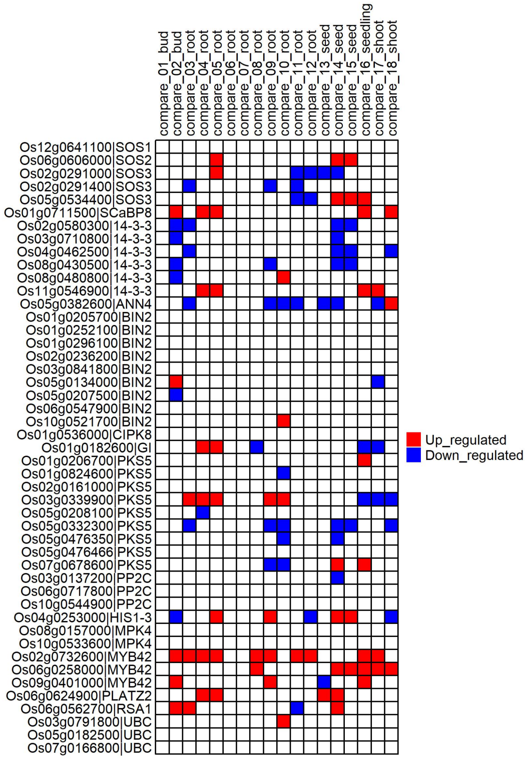

maize

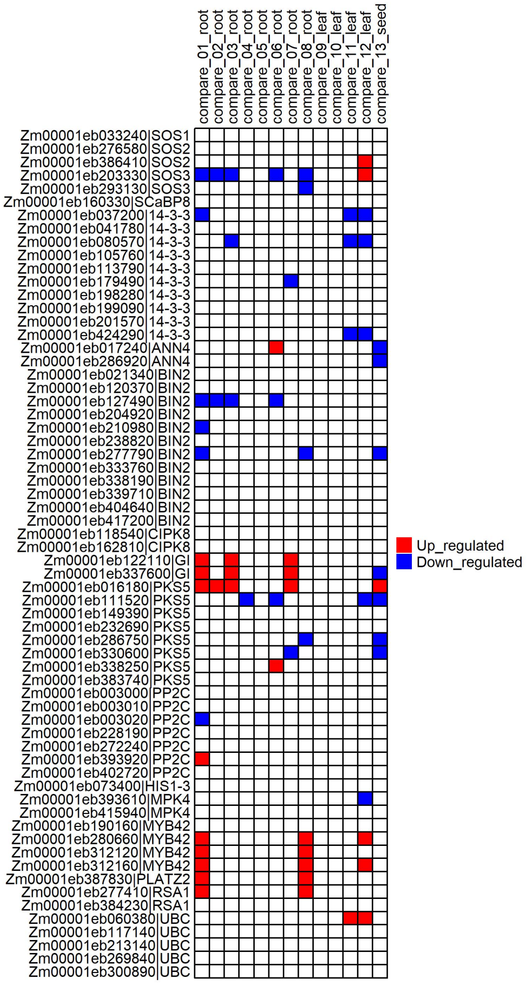

Supplementary Figure S13

A

MAPKKK
(Zosma03g00830, Zosma03g00810)

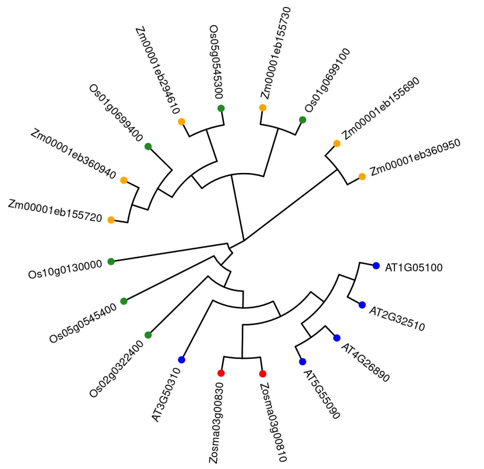

B

rice

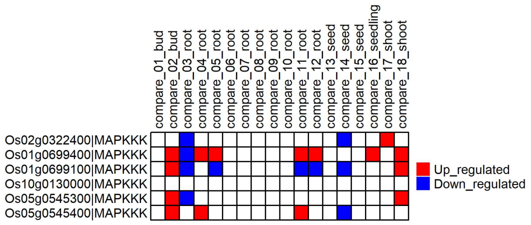

maize

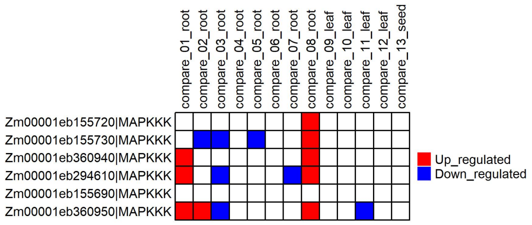

Supplementary Figure S14

A

bHLH

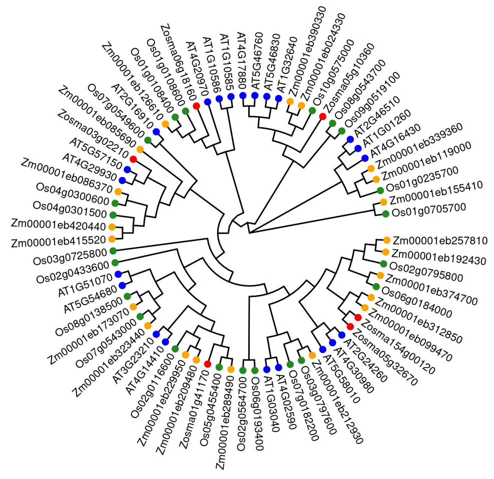

ERF

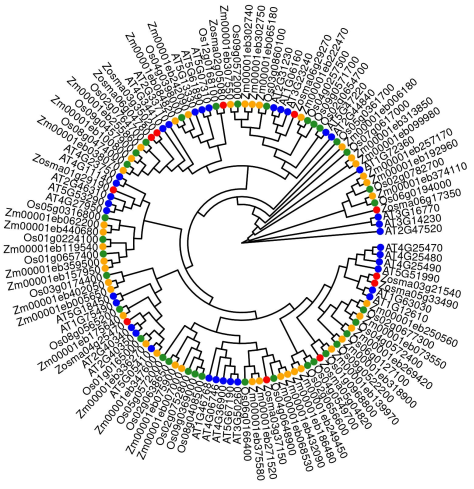

MYB

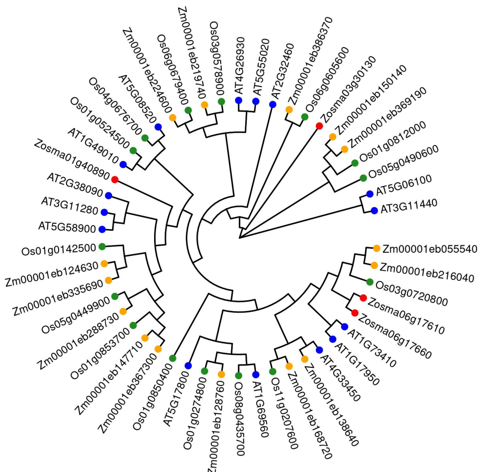

B

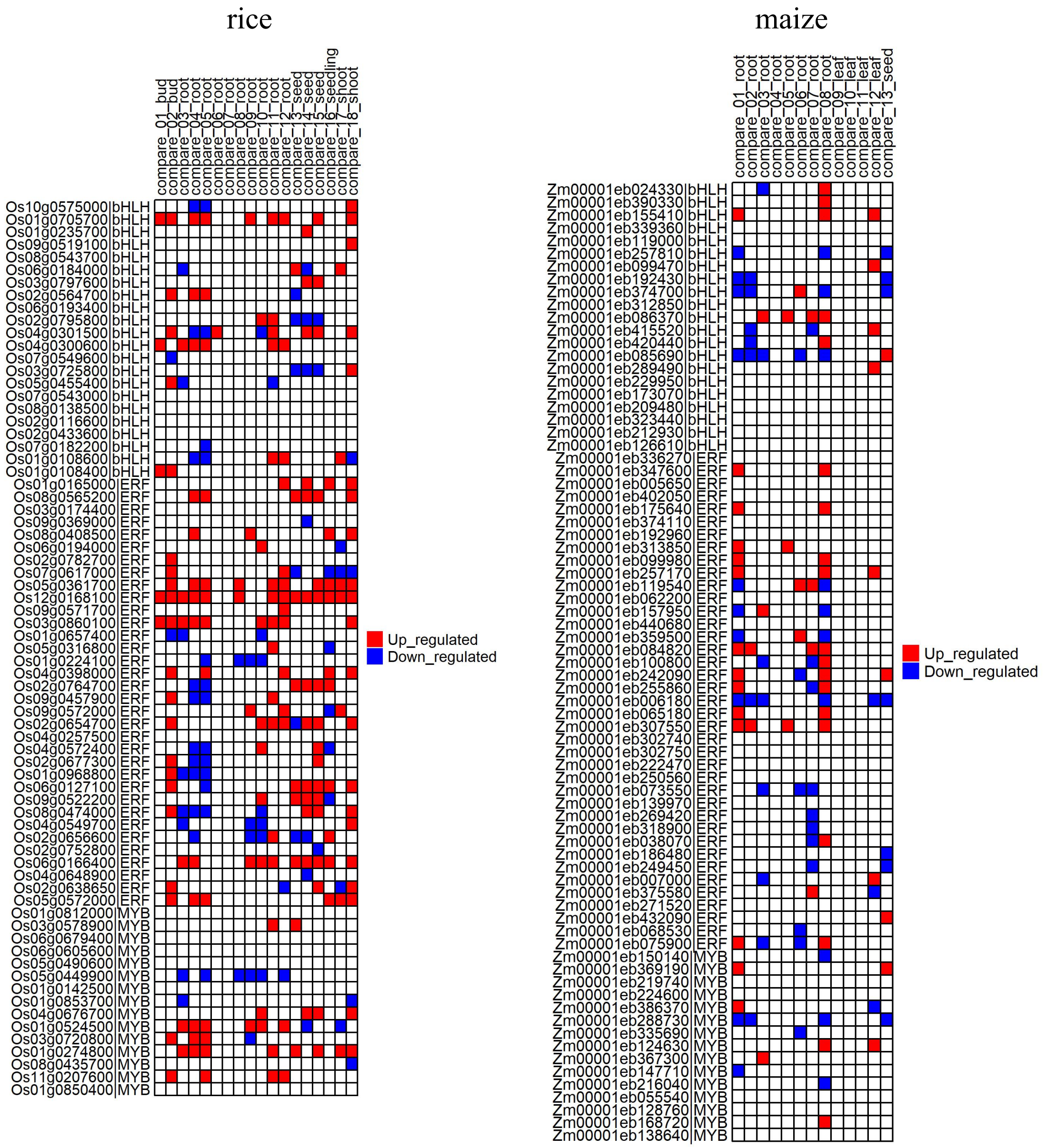

rice
compare_01_bud
compare_02_bud
compare_03_root
compare_04_root
compare_05_root
compare_06_root
compare_07_root
compare_08_root
compare_09_root
compare_10_root
compare_11_root
compare_12_root
compare_13_seed
compare_14_seed
compare_15_seed
compare_16_seedling
compare_17_shoot
compare_18_shoot
Os10g0575000|bHLH
Os01g0705700|bHLH
Os01g0235700|bHLH
Os09g0519100|bHLH
Os08g0543700|bHLH
Os06g0184000|bHLH
Os03g0797600|bHLH
Os02g0564700|bHLH
Os06g0193400|bHLH
Os02g0795800|bHLH
Os04g0301500|bHLH
Os04g0300600|bHLH
Os07g0549600|bHLH
Os03g0725800|bHLH
Os05g0455400|bHLH
Os07g0543000|bHLH
Os08g0138500|bHLH
Os02g0116600|bHLH
Os02g0433600|bHLH
Os07g0182200|bHLH
Os01g0108600|bHLH
Os01g0108400|bHLH
Os01g0165000|ERF
Os08g0565200|ERF
Os03g0174400|ERF
Os09g0369000|ERF
Os08g0408500|ERF
Os06g0194000|ERF
Os02g0782700|ERF
Os07g0617000|ERF
Os05g0361700|ERF
Os12g0168100|ERF
Os09g0571700|ERF
Os03g0860100|ERF
Os01g0657400|ERF
Os05g0316800|ERF
Os01g0224100|ERF
Os04g0398000|ERF
Os02g0764700|ERF
Os09g0457900|ERF
Os09g0572000|ERF
Os02g0654700|ERF
Os04g0257500|ERF
Os04g0572400|ERF
Os02g0677300|ERF
Os01g0968800|ERF
Os06g0127100|ERF
Os09g0522200|ERF
Os08g0474000|ERF
Os04g0549700|ERF
Os02g0656600|ERF
Os02g0752800|ERF
Os06g0166400|ERF
Os04g0648900|ERF
Os02g0638650|ERF
Os05g0572000|ERF
Os01g0812000|MYB
Os03g0578900|MYB
Os06g0679400|MYB
Os06g0605600|MYB
Os05g0490600|MYB
Os05g0449900|MYB
Os01g0142500|MYB
Os01g0853700|MYB
Os04g0676700|MYB
Os01g0524500|MYB
Os03g0720800|MYB
Os01g0274800|MYB
Os08g0435700|MYB
Os11g0207600|MYB
Os01g0850400|MYB
Up_regulated
Down_regulated
maize
compare_01_root
compare_02_root
compare_03_root
compare_04_root
compare_05_root
compare_06_root
compare_07_root
compare_08_root
compare_09_leaf
compare_10_leaf
compare_11_leaf
compare_12_leaf
compare_13_seed
Zm00001eb024330|bHLH
Zm00001eb390330|bHLH
Zm00001eb155410|bHLH
Zm00001eb339360|bHLH
Zm00001eb119000|bHLH
Zm00001eb257810|bHLH
Zm00001eb099470|bHLH
Zm00001eb192430|bHLH
Zm00001eb374700|bHLH
Zm00001eb312850|bHLH
Zm00001eb086370|bHLH
Zm00001eb415520|bHLH
Zm00001eb420440|bHLH
Zm00001eb085690|bHLH
Zm00001eb289490|bHLH
Zm00001eb229950|bHLH
Zm00001eb173070|bHLH
Zm00001eb209480|bHLH
Zm00001eb323440|bHLH
Zm00001eb212930|bHLH
Zm00001eb126610|bHLH
Zm00001eb336270|ERF
Zm00001eb347600|ERF
Zm00001eb005650|ERF
Zm00001eb402050|ERF
Zm00001eb175640|ERF
Zm00001eb374110|ERF
Zm00001eb192960|ERF
Zm00001eb313850|ERF
Zm00001eb099980|ERF
Zm00001eb257170|ERF
Zm00001eb119540|ERF
Zm00001eb062200|ERF
Zm00001eb157950|ERF
Zm00001eb440680|ERF
Zm00001eb359500|ERF
Zm00001eb084820|ERF
Zm00001eb100800|ERF
Zm00001eb242090|ERF
Zm00001eb255860|ERF
Zm00001eb006180|ERF
Zm00001eb065180|ERF
Zm00001eb307550|ERF
Zm00001eb302740|ERF
Zm00001eb302750|ERF
Zm00001eb222470|ERF
Zm00001eb250560|ERF
Zm00001eb073550|ERF
Zm00001eb139970|ERF
Zm00001eb269420|ERF
Zm00001eb318900|ERF
Zm00001eb038070|ERF
Zm00001eb186480|ERF
Zm00001eb249450|ERF
Zm00001eb007000|ERF
Zm00001eb375580|ERF
Zm00001eb271520|ERF
Zm00001eb432090|ERF
Zm00001eb068530|ERF
Zm00001eb075900|ERF
Zm00001eb150140|MYB
Zm00001eb369190|MYB
Zm00001eb219740|MYB
Zm00001eb224600|MYB
Zm00001eb386370|MYB
Zm00001eb288730|MYB
Zm00001eb335690|MYB
Zm00001eb124630|MYB
Zm00001eb367300|MYB
Zm00001eb147710|MYB
Zm00001eb216040|MYB
Zm00001eb055540|MYB
Zm00001eb128760|MYB
Zm00001eb168720|MYB
Zm00001eb138640|MYB
Up_regulated
Down_regulated

Supplementary Figure S15

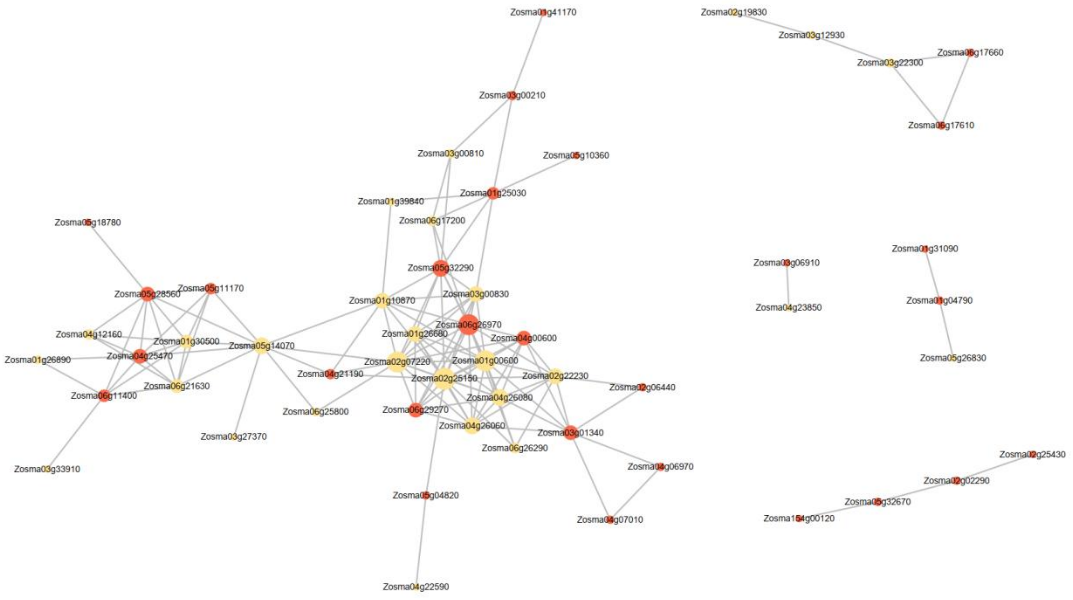

Supplementary Figure S16

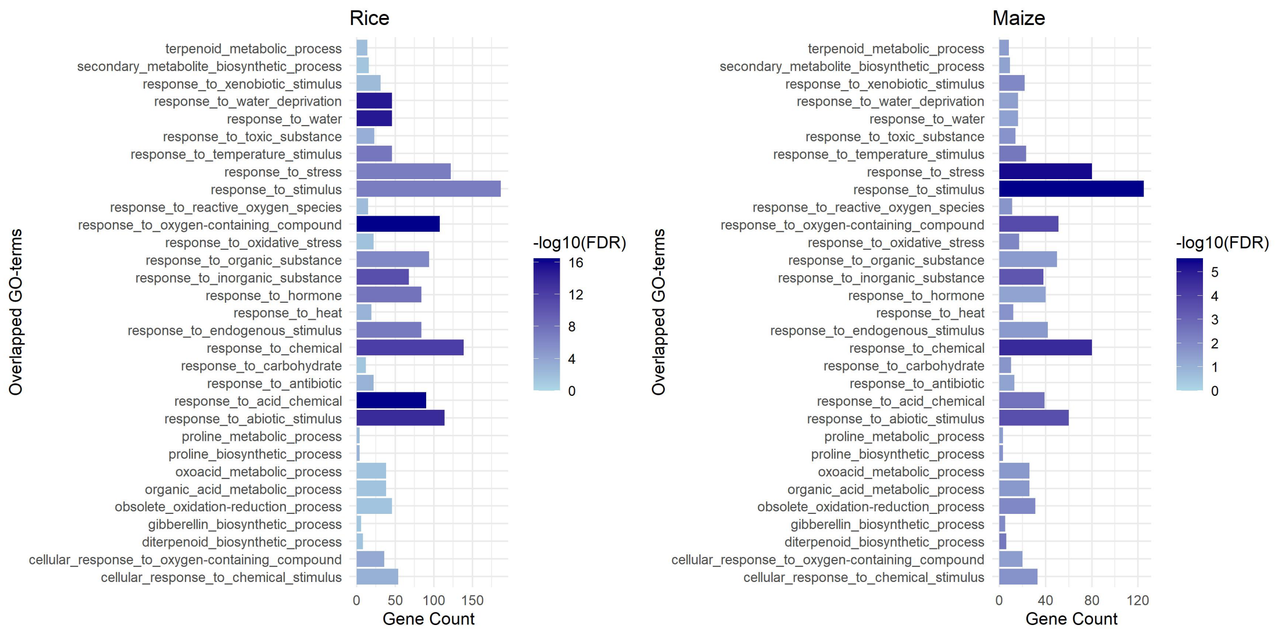

Supplement: Supplementary file 2 [file DataSheet1.pdf]
